# Controlling the confounding effect of metabolic gene expression to identify actual metabolite targets in microsatellite instability cancers

**DOI:** 10.1186/s40246-023-00465-9

**Published:** 2023-03-06

**Authors:** Chung-I. Li, Yu-Min Yeh, Yi-Shan Tsai, Tzu-Hsuan Huang, Meng-Ru Shen, Peng-Chan Lin

**Affiliations:** 1 Department of Statistics, National Cheng Kung University, Tainan, 704 Taiwan; 2 Department of Oncology, National Cheng Kung University Hospital, College of Medicine, National Cheng Kung University, 138 Sheng-Li Road, Tainan, Taiwan; 3 Department of Medical Imaging, National Cheng Kung University Hospital, College of Medicine, National Cheng Kung University, Tainan, 704 Taiwan; 4 Institute of Data Science, National Cheng Kung University, Tainan, 704 Taiwan; 5 Institute of Clinical Medicine, National Cheng Kung University Hospital, College of Medicine, National Cheng Kung University, Tainan, 704 Taiwan; 6 Department of Obstetrics and Gynecology, National Cheng Kung University Hospital, College of Medicine, National Cheng Kung University, Tainan, 704 Taiwan; 7 Department of Pharmacology, National Cheng Kung University Hospital, College of Medicine,, National Cheng Kung University, Tainan, 704 Taiwan; 8 Department of Genomic Medicine, National Cheng Kung University Hospital, College of Medicine, National Cheng Kung University, Tainan, 704 Taiwan

**Keywords:** Metabolomic, Metabolic gene, CATCH model, Confounding effect, Metabolic targets, Microsatellite instability

## Abstract

**Background:**

The metabolome is the best representation of cancer phenotypes. Gene expression can be considered a confounding covariate affecting metabolite levels. Data integration across metabolomics and genomics to establish the biological relevance of cancer metabolism is challenging. This study aimed to eliminate the confounding effect of metabolic gene expression to reflect actual metabolite levels in microsatellite instability (MSI) cancers.

**Methods:**

In this study, we propose a new strategy using covariate-adjusted tensor classification in high dimensions (CATCH) models to integrate metabolite and metabolic gene expression data to classify MSI and microsatellite stability (MSS) cancers. We used datasets from the Cancer Cell Line Encyclopedia (CCLE) phase II project and treated metabolomic data as tensor predictors and data on gene expression of metabolic enzymes as confounding covariates.

**Results:**

The CATCH model performed well, with high accuracy (0.82), sensitivity (0.66), specificity (0.88), precision (0.65), and F1 score (0.65). Seven metabolite features adjusted for metabolic gene expression, namely, 3-phosphoglycerate, 6-phosphogluconate, cholesterol ester, lysophosphatidylethanolamine (LPE), phosphatidylcholine, reduced glutathione, and sarcosine, were found in MSI cancers. Only one metabolite, Hippurate, was present in MSS cancers. The gene expression of phosphofructokinase 1 (*PFKP*), which is involved in the glycolytic pathway, was related to 3-phosphoglycerate. *ALDH4A1* and *GPT2* were associated with sarcosine. LPE was associated with the expression of *CHPT1*, which is involved in lipid metabolism. The glycolysis, nucleotide, glutamate, and lipid metabolic pathways were enriched in MSI cancers.

**Conclusions:**

We propose an effective CATCH model for predicting MSI cancer status. By controlling the confounding effect of metabolic gene expression, we identified cancer metabolic biomarkers and therapeutic targets. In addition, we provided the possible biology and genetics of MSI cancer metabolism.

**Supplementary Information:**

The online version contains supplementary material available at 10.1186/s40246-023-00465-9.

## Background

Microsatellite instability (MSI) results in the frequent occurrence of short tandem repeats in the cancer genome when there is a deficit in DNA mismatch repair (MMR) genes. In addition to DNA repair mechanisms, somatic genetic alterations such as tumor suppressor genes may accumulate in MSI malignancies [1]. Clinically, several malignancies, notably endometrial, upper gastrointestinal (GI), colorectal, cervical, and prostate cancers, express MSI [2, 3]. It is a valuable marker for predicting immunotherapy responses in any type of cancer [3, 4]. However, MSI cancer patients show a variety of biological mechanisms of immune resistance to immunotherapy. Intrinsic resistance to immunotherapy is commonly attributed to host genetic alterations, immune response, and tumor metabolism [5]. The relationship between drug resistance and MSI cancer metabolites can be understood by investigating tumor metabolites and MSI status. High-throughput metabolomics has been used to identify new diagnostic biomarkers and therapeutic targets for various cancers [6]. In MSI cancers, plasma metabolites can be used as biomarkers for diagnosis, recurrence surveillance, and treatment response monitoring [7].

Metabolomics is the best representation of molecular phenotypes and the final step in the omics cascade. Gene alterations have been proposed to significantly affect metabolite levels. For example, some genetic loci have been identified to correlate with specific metabolic phenotypes using quantitative trait locus (QTL) mapping [8]. Metabolites and gene expression of metabolic enzymes are two fundamental biological components of metabolic pathways. Metabolites represent a variety of upstream biological signals at the functional genomic level, such as the transcriptome [9]. As a result of epigenetic modifications, metabolic enzyme genes expressed differently might contribute to metabolic reprogramming, which is necessary for glucose metabolism, lipid metabolism, and amino acid metabolism [10]. In addition, research has indicated the direct involvement of the metabolome in genome regulation [11]. Metabolomics also serves as an input that influences genomic alteration to form a feedback loop [12, 13]. There are interactions between metabolites and gene expression [14, 15].

Covariate adjustment has been described in previous metabolite studies as a method for reducing confounders [16]. Metabolism is altered by many factors, such as genetics, disease status, and the environment. Gene expression of metabolic enzymes is a confounding covariate that affects metabolite levels in different cancer types [8]. In our study, genetic alterations such as gene mutations and gene expression affected metabolite levels and MSI cancer status. By controlling the confounding effects, we can identify the actual metabolite biomarkers and cancer metabolism. Integrating information from metabolomic predictors and genomic confounding covariates to predict MSI cancer status is challenging. In multiomics analyses, various omics datasets have been used to investigate the underlying biological mechanisms of diseases [17]. Most studies use a simultaneous integration and linear regression approach to interpret multiomics data [17, 18]. For example, MetaboAnalyst v5.0 [18], a metabolite analysis software, uses a linear regression model to adjust for individual features such as age, sex, and batch variables. Multiomics data are integrated and interpreted using joint pathway analysis. Random forest (RF) is used to integrate multiple factors simultaneously [17]. However, the current interface does not allow the incorporation of high-dimensional tensor predictors and confounding covariates to achieve the best possible classification.

To integrate metabolomic and genomic data, we propose a novel strategy using covariate-adjusted tensor classification in high dimensions (CATCH) models [19]. The main goal of this study was to minimize the impact of confounding covariates in identifying actual metabolism in MSI or microsatellite stability (MSS) cancers. Metabolites and gene expression may interact with each other. Moreover, the metabolome is closely related to the phenotype. Thus, we treated metabolomic data as tensor predictors and genomic data as confounding covariates. Unlike typical metabolomics analysis [18, 20, 21], the CATCH model uses the tensor regression approach to define the relationship between the metabolomic and genomic data. This research reveals the adjusted metabolite features, the predictive performance of the CATCH model, and the relationships between metabolite features and metabolic genes. Finally, we discuss metabolic pathways in MSI and MSS cancers.

## Methods

### Cancer cell lines, gene alterations, MSI cancer status, and metabolites

Metabolomic and genomic data were collected from phase II of the Cancer Cell Line Encyclopedia (CCLE) project [22–24]. CCLE data, including RNA expression and genetic mutation data for over 1000 cancer cell lines across 20 major cancer types, are publicly accessible. Emerging next-generation sequencing (NGS) technology was applied to RNA expression data. Data on cancer cell lines were obtained from the Cancer Dependency Map Project (DepMap) (https://depmap.org/portal/download/custom/). The cancer cell lineages (from the sample information file), *APC* mutations (from the mutation file), and RNA gene expression data (from the expression 2022Q2 public file) were found in the DepMap 22Q2 data release (accessed on May 26, 2022). MSI cancer status was determined using NGS and polymerase chain reaction (PCR)-based phenotyping [24]. We downloaded the MSI/MSS cancer status and *TP53* mutation data from E. M. Chan’s research project [24]. A total of 928 cell lines were analyzed by liquid chromatography‒mass spectrometry (LC‒MS). Metabolite profiling revealed 225 metabolites, including 124 polar and 101 lipid species. The CCLE 2019 metabolomics dataset (clean and imputed) was used for further analysis [25].

### MSI and MSS cancer cell line matching

A total of 75 MSI and 827 MSS cancer cell lines were identified. To reduce bias between metabolite data from MSI and MSS cancer cell lines, the MSS cell lines were randomly sampled to match *APC* and *TP53* mutations as well as cancer cell lineages. Finally, 225 MSI cancer cell lines were selected for subsequent analysis.

### CATCH model

We used the CATCH model proposed by Pan et al. [19] to develop a classifier for predicting the MSI cancer status from metabolite profiling and gene expression data. In statistics, a confounding covariate, e.g., metabolic gene expression, is a variable that influences both the dependent variable (MSI status) and independent variable (metabolites, as Additional file 1: Fig. S1). The CATCH model can be used to predict particular classes by controlling the confounding effects. We considered the gene expression of metabolic enzymes as a confounding covariate and used the CATCH model to predict the MSI status. The CATCH model is a classifier based on Bayes' rule and is defined as follows:

$$\hat{Y} = {\text{arg}}\mathop {\max }\limits_{k = 1,2} {\text{Pr}}\left\{ {a_{k} + \gamma_{k}^{T} G + \left\langle {B,M^{adj} } \right\rangle } \right\},$$

where $\widehat{Y}$ is the predictor for MSI cancer status (1 for MSS; 2 for MSI), ${\mathrm{M}}^{\mathrm{adj}}= M-{\alpha }_{(M+1)}\overline{\times }G$ represents metabolite profiling data adjusted by the gene expression level G, and M represents the original metabolite data profiling. The coefficient $\alpha$ is used to quantify the relationship between metabolite levels and gene expression, while coefficient $B$ represents the direct effect of metabolite levels on MSI cancer status. The coefficients $\{B$, $\alpha \}$ and ${\mathrm{M}}^{\mathrm{adj}}$ are critical and can guide clinicians in interpreting the results obtained from the CATCH model. Here, we explain how to utilize this information to identify metabolite biomarkers for, biological relevance of, and potential therapeutic targets in MSI cancers. Because the CATCH method involves a variable selection algorithm, a typical data standardization procedure was used to transform the metabolite data to comparable scales. The datasets and source code are available at https://github.com/H24061024/microsatellite-instability-cancers.

### Data visualization tools

The R package pheatmap (version 1.0.12) was used to draw a heatmap to visualize the correlation between metabolite features and metabolic gene expression. A boxplot was created using the R package ggplot2 (version 3.3.6) to visually assess the differences in non-adjusted, standardized, and CATCH-adjusted metabolite data between MSI and MSS cancers. Additionally, we performed the Wilcoxon test to evaluate the statistical significance of the non-adjusted, standardized, and CATCH-adjusted metabolite data between MSI and MSS cancers. A *p* value greater than 0.05 was considered statistically significant.

### Integration models for metabolomic and genomic data

**Fig. 1:**
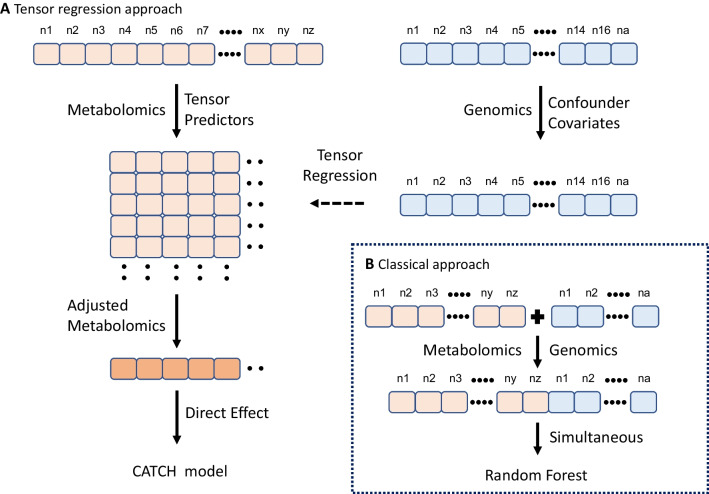
Integration models for metabolomic and genomic data. **A** Tensor regression approach. Tensor datatype was used to represent the metabolomic data for predicting MSI cancer status. The genomic data were treated as confounding covariates. The adjusted metabolomic and genomic data were correlated using a tensor regression model. **B** Classical approach. The metabolomic and genomic data were integrated with a simultaneous approach for classification and correlated using a linear regression model

Two conceptual approaches incorporating metabolomic and genomic data are shown in Fig. 1. CATCH models were employed in this study to predict MSI and MSS cancer statuses. Metabolomic data were treated as tensor predictors, whereas genomic data were treated as confounding covariates (Fig. 1A). To classify MSI and MSS cancer status, we adjusted the metabolomic data with genomic data and quantified the direct impacts on the outcome. A tensor regression model was used to determine the relationship between metabolomic and genomic data. For comparison, we used a classical machine learning technique, RF, to simultaneously integrate metabolomic and genomic data (Fig. 1B) [26].

### Databases and metabolic pathway analysis

Public databases for metabolite information and metabolic pathways were used. PubChem is an open database maintained by the National Institutes of Health (NIH) that allows users to search for metabolites by name and identify their chemical and physical properties along with other information [27]. The Human Metabolome Database (HMDB) was used for metabolite information, including chemical, clinical, molecular biology, and biochemical data [28]. The metabolic pathway of the biological system was analyzed using the Kyoto Encyclopedia of Genes and Genomes (KEGG) database [29].

### Analysis of plasma metabolites in MSI cancer patients

The case study was approved by the Institutional Review Board (IRB) of the National Cheng Kung University Hospital (NCKUH) (A-ER-103–395, B-ER-110–342, and B-ER-110–418), and the healthy control study was approved by the IRB of NCKUH (B-ER-110–442). The study was conducted in accordance with the Declaration of Helsinki. We used LC‒MS for amino acid and related amine analysis and nuclear magnetic resonance (NMR) for nonamine metabolite analysis. Plasma was collected from a patient with MSI cancer and healthy control subjects. Protein precipitation using methanol was carried out as described by Gowda [30]. NMR experiments were conducted at 298 K on a Bruker Avance III 600 MHz spectrometer (Billerica, MA, USA) equipped with a triple-inverse probe and a Z-gradient. CPMG (Carr − Purcell − Meiboom − Gill) pulse sequences and presaturation for water suppression were used for ^1^H 1D NMR experiments. For the LC‒MS-based metabolomics study, the amino acid derivatives were prepared according to the methods described in the Kairos™ amino acid kit manual of Waters™ (Milford, MA, USA). The precipitated samples were derivatized using the AccQ Tag™ Derivatization kit (Waters Corporation). The LC‒MS system consisted of an ACQUITY® UPLC® H-Class Plus System (Waters Corporation) and an ACQUITY® QDa® Mass Detector (mass spectrometry detector; Waters Corporation) equipped with an electrospray ionization interface. Ultra-performance liquid chromatography‒mass spectrometry (UPLC-QDa, UPLC‒MS) was used for analysis. A CORTECS® UPLC® C18 column (2.1 mm × 150 mm, 1.6 μm particle size) was used for compound separation. Information regarding the identified metabolites was confirmed in our preliminary results by matching the LC‒MS or NMR information with the analysis of various metabolites of the internal standard.

## Results

### Characteristics of cancer cell lines and matching

The metabolite and gene expression data of 902 cancer cell lines were identified in the DepMap database. Of these cancer cell lines, 827 were associated with MSS cancers, whereas 75 were associated with MSI cancers. We selected and matched cell lines on a 1:3 basis for both MSI and MSS cancers (Additional file 4: Table S1). *APC* mutations, *TP53* mutations, and cancer cell lines from GI, breast, gynecologic (GYN), hematologic (Hema), genitourinary (GU), and other cancer cell lineages were used to match MSI (*n* = 75) and MSS (*n* = 225) cancers. Additional file 4: Table S1 presents comparisons of MSI- and MSS-matched cancer cell lines. The percentage of *APC* mutations was 25.3% in MSI cancers and 20.4% in MSS cancers. No discernible distinction could be made between MSI and MSS cancers in terms of the clinical characteristics of *TP53* mutations and cancer cell lineages. There were 32% *TP53* mutations in both MSI and MSS cancers. Regarding cancer lineages, GI cancers accounted for 33.3%, breast and gynecological cancers accounted for 34.7%, and hematological cancers accounted for 17.3% of cancer cell lines. Based on data from 300 cancer cell lines, CATCH model analysis was applied to adjust metabolite data using gene expression data as covariates.

### Identification of adjusted metabolite features affecting MSI cancer status by the CATCH model

**Fig. 2:**
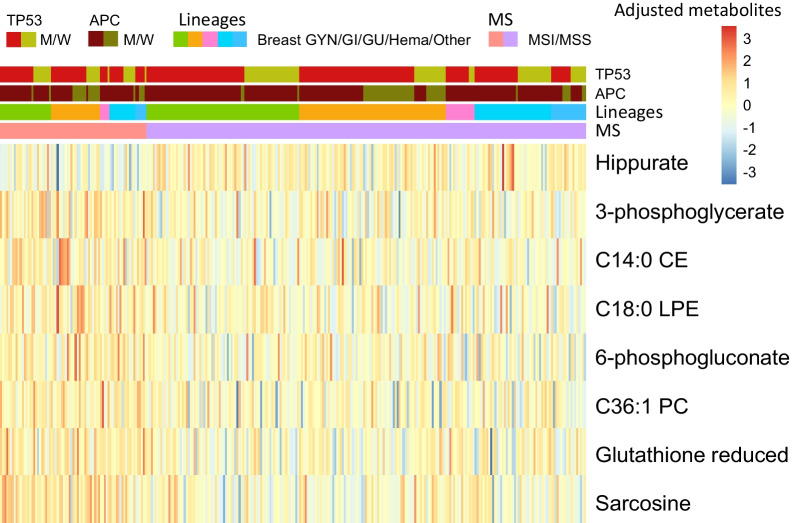
Adjusted metabolite features with confounding covariates in MSI and MSS cancers. The heatmap illustrates the relationship between adjusted metabolite features and microsatellite instability (MSI)/microsatellite stable (MSS) cancer status. In the CATCH model, the Y-axis displays adjusted metabolite features and levels. Eight crucial adjusted metabolite features were found to distinguish between MSI and MSS cancers. The cancer cell lineages (gastrointestinal (GI), breast, and gynecologic (GYN), hematologic (Hema), genitourinary (GU), and others), MSI/MSS cancer status, and *APC* and *TP53* mutations (mutation/wild type: M/W) are displayed on the X-axis. Positive values for adjusted metabolite features suggest a stronger association with MSI cancers, while negative values represent a strong association with MSS cancer. The eight different metabolite features were Hippurate, 3-phosphoglycerate, cholesterol ester (CE, C14:0), lysophosphatidylethanolamine (LPE, C18:0), 6-phosphogluconate, phosphatidylcholine (PC, C36:1), reduced glutathione (GSH), and sarcosine

To use the CATCH method for predicting MSI cancer status, 225 metabolite data points were considered $25\times 25$ tensor data, and 87 metabolic genes were considered confounding covariates (Fig. 1A). The 87 metabolic genes were selected from four major metabolic pathways associated with 225 metabolites (Additional file 5: Tables S2 and Additional file 6: Table S3), namely, the amino acid, carbohydrate, lipid, and nucleotide metabolic pathways [31]. The eight most significant adjusted metabolite features were selected based on the variable selection algorithm in the CATCH model for predicting the MSI cancer status (Additional file 7: Table S4 and Fig. 2). The adjusted metabolite features distinguished MSI from MSS cancer. The direct effect, coefficient $B$ in the CATCH model, on the MSI cancer status ranged from − 0.17–0.56 (Table 1).

**Table 1:** Direct effect of adjusted metabolite features

Features	Coefficient
Hippurate	− 0.17
3-phosphoglycerate	0.01
C14:0 cholesterol ester (CE)	0.01
C18:0 lysophosphatidylethanolamine (LPE)	0.07
6-phosphogluconate	0.09
C36:1 phosphatidylcholine (PC)	0.10
Glutathione reduced	0.20
Sarcosine	0.56

Positive coefficient values implied that the metabolite features were more relevant to the MSI cancer status. Seven adjusted metabolite features were present in MSI cancer cell lines, namely, 3-phosphoglycerate, cholesterol ester (CE, C14:0), lysophosphatidylethanolamine (LPE, C18:0), 6-phosphogluconate, phosphatidylcholine (PC, C36:1), reduced glutathione (GSH), and sarcosine. All had a positive relationship with MSI cancer.

3-phosphoglycerate is related to cellular energy. The glycolytic intermediate 3-phosphoglycerate is a source of sarcosine and serine. The oncometabolite sarcosine has been associated with invasive prostate cancer cells [32]. 6-phosphogluconate affects nucleotide metabolism, which aids cell growth. CEs, LPE, and PC are also related to lipid metabolism in cancer [33]. Glutathione is associated with the survival of cancer cells through reactive oxygen species (ROS) mechanisms. Clinical studies have also linked glutathione to chemotherapy resistance [34].

If the coefficient value was negative, then the metabolite feature exhibited greater relevance to MSS cancer. One metabolite feature, Hippurate, was associated with MSS cancer. Based on the CATCH model, we demonstrated the direct effect of adjusted metabolites on the prediction of MSI cancer status.

### Performance of the CATCH model

Using metabolomic and genomic data, we compared the performance of the CATCH model with that of RF, the most common classification algorithm in machine learning. To evaluate the performance, we randomly split the entire dataset into training and testing datasets. MSI and MSS cancer cell lines were maintained at a 1:3 ratio throughout the training and testing datasets. The training dataset contained 90% of the entire data, whereas the testing dataset contained 10%. The splitting process was run for 100 iterations, and the average performance metrics were calculated. Table 2 shows the performance of the RF and CATCH models. The CATCH model performed well, with high accuracy (0.82), sensitivity (0.66), specificity (0.88), precision (0.65), and F1 score (0.65). For RF, a simultaneous approach was used to predict MSI cancer status. The RF model had an accuracy of 0.77, sensitivity of 0.10, specificity of 0.99, precision of 0.81, and F1 score of 0.26. The CATCH model was more accurate in classifying MSI and MSS cancer status than the RF model in terms of accuracy and F1 score.

**Table 2:** Performance of CATCH and random forest models

Methods	Accuracy	Sensitivity	Specificity	Precision	F1 score
Random forest	0.767	0.096	0.991	0.805	0.258
CATCH	0.824	0.656	0.880	0.654	0.647

*CATCH* Covariate-adjusted, proposed tensor classification in high dimensions

### The significance of metabolite data with or without adjustment

To better understand the confounding effects of gene expression covariates on metabolite features, we compared their significance between non-adjusted and CATCH-adjusted metabolite data. Additional file 2: Fig. S2 displays a boxplot comparing the non-adjusted, standardized, and CATCH-adjusted metabolite data between MSI and MSS cancers. Considering the confounding covariates of metabolic genes, we obtained eight adjusted metabolite features that were strongly correlated with MSI and MSS cancers (*p* < 0.05, Supplementary Fig. S2). Supplementary Fig. S2 shows that three metabolite features, namely, 3-phosphoglycerate (non-adjusted and standardized, *p* = 0.855), LPE (C18:0) (non-adjusted and standardized, *p* = 0.056), and GSH (non-adjusted and standardized, *p* = 0.25), were initially not correlated with MSI and MSS cancers, but after adjustment, they had a significant correlation with MSI cancers (*p* < 0.001). Hippurate, CE (C14:0), 6-phosphogluconate, PC (C36:1), and sarcosine were five non-adjusted and standardized metabolite features that were substantially associated with MSI or MSS cancers (*p* < 0.05).

**Fig. 3:**
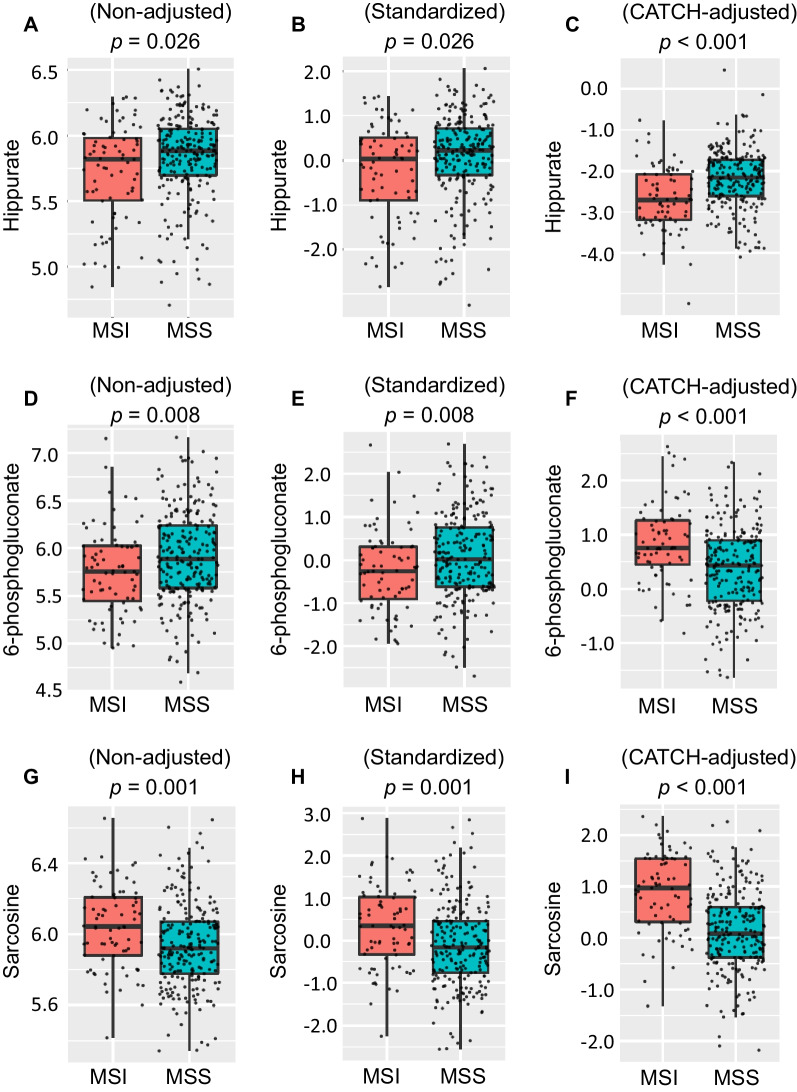
CATCH model-adjusted versus non-adjusted metabolite data in MSI and MSS cancers. Boxplot comparing the differences among the non-adjusted, standardized, and CATCH-adjusted metabolite levels as well as the *p* value in MSI and MSS cancers. Boxplots of non-adjusted, standardized, CATCH-adjusted Hippurate levels and *p* values are shown in **A, B,** and** C**, respectively. Boxplots of non-adjusted, standardized, CATCH-model-adjusted 6-phosphogluconate levels and *p* values are shown in **D** and** F**, respectively. Boxplots of non-adjusted, standardized, CATCH model-adjusted sarcosine levels and *p* values are shown in** G, H,** and** I**, respectively

Hippurate, for example, had a higher level in MSS cancers (non-adjusted and standardized, *p* = 0.026) (Fig. 3A, 3B). After adjustment, it was more significantly associated with MSS cancers (CATCH-adjusted, *p* value < 0.001) (Fig. 3C). Without adjustment for metabolic gene expression, the level of 6-phosphogluconate was negatively correlated with MSI cancers (non-adjusted and standardized, *p* value = 0.008) (Fig. 3D, 3E). In contrast, it was positively associated with MSI cancers after elimination of the confounding effect of metabolic gene expression (CATCH-adjusted, *p* value < 0.001) (Fig. 3F). Sarcosine had a higher value and was positively correlated with MSI cancers (non-adjusted and standardized, *p* value = 0.001) (Fig. 3G, 3H). After adjustment for metabolic gene expression, sarcosine was more significantly associated with MSI cancers (CATCH-adjusted, *p* < 0.001) (Fig. 3I).

### The relationship between adjusted metabolite features and metabolic genes

**Fig. 4:**
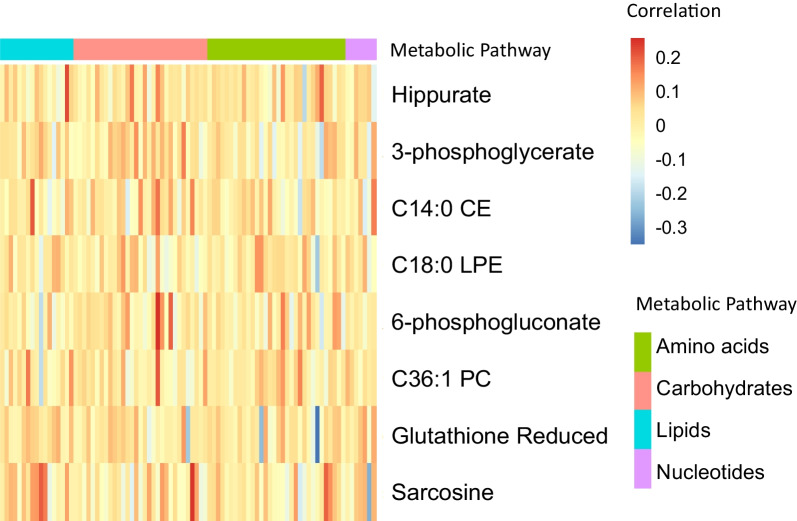
The relationships between adjusted metabolite features and metabolic gene expression. The correlation between eight significantly adjusted metabolite features and 87 metabolic genes was used to create a heatmap. The Y-axis displays eight adjusted metabolite features, including Hippurate, 3-phosphoglycerate, CE (C14:0), LPE (C18:0), 6-phosphogluconate, PC (C36:1), GSH, and sarcosine. On the X-axis, each metabolic pathway is represented by 87 metabolic genes, including amino acids, carbohydrates, lipids, and nucleotides

We quantified the relationship between adjusted metabolite levels and metabolic gene expression in cancer cell lines to identify the potential metabolic pathways in MSI cancers. The *α* coefficients are listed in Additional file 8: Table S5. In Fig. 4, we present a heatmap visualization based on eight adjusted metabolite features and 87 metabolic genes. Table 3 shows the eight adjusted metabolite features and metabolic genes in the same metabolic pathway. Hippurate is correlated with the expression of metabolic genes such as *QDPR*, *FAH*, *PAOX*, *MPST*, and *SLC7A5*, which are involved in the metabolism of amino acids and their derivatives. 3-phosphoglycerate is related to *ST3GAL2*, *PFKP*, *HS3ST1*, *HPSE*, and *PGM1* metabolic gene expression, which are involved in carbohydrate metabolism. LPE is linked to the expression of metabolic genes such as *PTGS1*, *CHPT1*, *SC5D*, *PLA2G3*, and *DHCR24*, which are involved in lipid metabolism. Sarcosine has been associated with *ALDH4A1*, *GPT2*, *AGMAT*, *ASL*, *AADAT*, and *MPST* metabolic gene expression involved in the metabolism of amino acids. By investigating the relationship between adjusted metabolite features and metabolic gene expression, we found potential biological relevance in cancer metabolic pathways.

**Table 3:** The relationship between adjusted metabolites and metabolic genes in the same metabolic pathway

Features	Metabolic pathway	Associated genes
Hippurate	Metabolism of amino acids	QDPR/FAH/PAOX/MPST/SLC7A5
3-phosphoglycerate	Metabolism of carbohydrates	ST3GAL2/PFKP/HS3ST1/HPSE/PGM1
C14:0 CE	Metabolism of lipids	AGPS/SGPP1/SQLE/EPHX2
C18:0 LPE	Metabolism of lipids	PTGS1/CHPT1/SC5D/PLA2G3/DHCR24
6-phosphogluconate	Metabolism of carbohydrates	B4GALT2/PFKFB2/PPP1R3C/SLC25A13/IDUA
C36:1 PC	Metabolism of lipids	CYP51A1/CERS6/PTGS1/PLA2G3/CERS4/SQLE
Glutathione reduced	Metabolism of amino acids	MRI1/FAH/GAMT/PAOX/CHDH/CDO1
Sarcosine	Metabolism of amino acids	ALDH4A1/GPT2/AGMAT/ASL/AADAT/MPST

*CE* Cholesterol ester, *LPE* Lysophosphatidylethanolamine, *PC* Phosphatidylcholine

### Cancer metabolism in MSI and MSS cancers

**Fig. 5:**
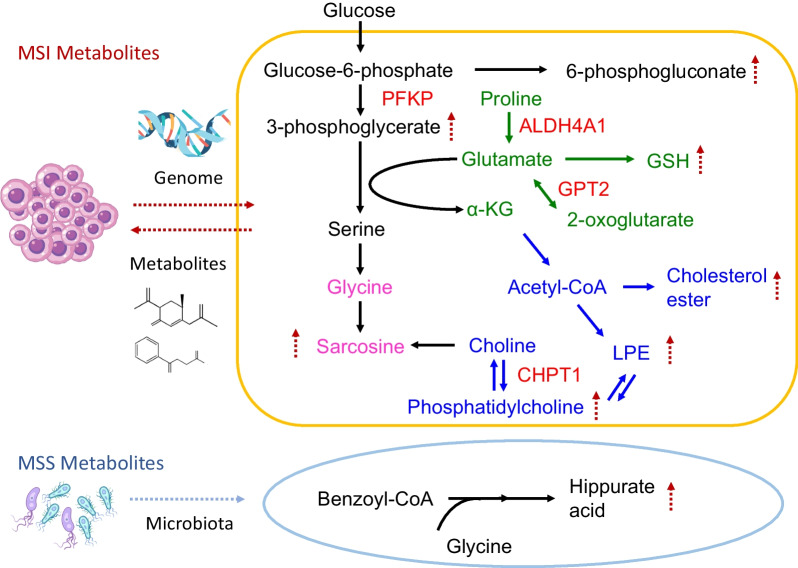
Metabolic pathways in MSI and MSS cancers. MSI cancer cells exhibit glycolytic metabolism, including the serine synthesis (sarcosine synthesis) and pentose phosphate (nucleotide synthesis) pathways. Sarcosine, 3-phosphoglycerate, and 6-phosphogluconate levels were elevated. Additionally, lipid metabolism and GSH synthesis were observed in MSI cancer metabolism. Levels of PC, LPE, CE, and GSH were elevated. Phosphofructokinase 1 (*PFKP*), *ALDH4A1*, *GPT2*, and *CHPT1* are involved in MSI cancer metabolism. These metabolic pathways promote cancer cell proliferation, energy production, and survival. DNA repair genetic mutations drive cancer metabolism, and sarcosine damages the DNA. Sarcosine and genomic alterations can regulate each other. In MSS cancers, environmental factors, such as the microbiota, may play a crucial role in Hippurate synthesis

Figure 5 displays the results of the metabolic pathway analysis using the HMDB and the KEGG databases [28, 29]. Cancer metabolism involves eight critical adjusted metabolites and four metabolic genes. Metabolic pathways are related to glycolysis, nucleotide, glutamate, and lipid metabolism. In MSI cancers, the four major metabolic pathways for cancer metabolism are the serine synthesis pathway (3-phosphoglycerate and sarcosine), pentose phosphate pathway (6-phosphogluconate), glutamate pathway (GSH), and lipid metabolism pathway (CE, LPE, and PC). After integrating the adjusted metabolite features and metabolic genes in the glycolytic and glutamate metabolic pathways, we found that 3-phosphoglycerate increased with phosphofructokinase 1 (*PFKP*) metabolic gene expression in the CATCH model. Sarcosine was associated with the expression of *ALDH4A1* and *GPT2* metabolic genes (Table 3). Proline is converted to glutamate through the expression of the *ALDH4A1* metabolic gene. *GPT2* metabolic gene expression is involved in the conversion of 2-oxoglutarate to glutamate. These findings suggest that an increase in sarcosine levels may occur due to glycolytic and glutamate metabolism. The conversion of choline to PC, which increases LPE metabolism, involves *CHPT1* metabolic gene expression. These results indicate that dysregulation of *PFKP*, *ALDH4A1*, *GPT2,* and *CHPT1* metabolic gene expression may lead to cancer metabolism in MSI cancer cell lines.

## Discussion

To identify more metabolite biomarkers and therapeutic targets for MSI cancers, we developed a new strategy using the tensor regression approach. Our results highlight the following crucial points: (i) we integrated metabolite and metabolic gene data using a powerful CATCH model for predicting MSI cancer status, (ii) seven adjusted metabolite features were identified for MSI metabolite biomarkers, and one metabolite feature was identified for MSS cancers, (iii) the relationship between adjusted metabolite features and metabolic genes was quantified, and (iv) we established metabolic pathways related to glycolysis, nucleotide, glutamate, and lipid metabolism in MSI cancers. These results provide information on cancer metabolism and possible therapeutic targets for MSI and MSS cancers.

The small-molecule compounds present in biological samples constitute the metabolome. Since 2007, the Human Metabolome Database (HMDB) has provided comprehensive metabolite properties, including biological, physiological, and chemical information. Recently, HMDB 5.0 (https://hmdb.ca) released 1,581,537 unannotated derivatized metabolite compounds and 217,920 annotated metabolite compounds [28]. The tensor approach is a useful method for managing high-dimensional metabolomic data. The tensor-based covariate approach has recently been used in multiomics data analysis [35] to predict continuous outcomes. In contrast, our model demonstrated the application of tensor predictors to a high-dimensional metabolome for predicting binary outcomes. Unlike classical methods using a simultaneous approach, only the direct effect of adjusted metabolite features was identified using the CATCH model (Fig. 1). The actual biological relevance can also be established by quantifying the relationship between the metabolomic and genomic data.

Metabolic gene expression affects the relationship between metabolite features and MSI cancer status. The primary goal of our study was to eliminate the impact of confounding covariates to reflect actual metabolite levels. Genetic expression and mutation may also regulate cancer-related metabolites. DNA damage signaling, such as the *TP53* mutation, can control glycolysis [36]. As a result of *APC* mutation, energy metabolic pathways may change, which also aids in cancer growth. The APC-WNT signaling pathway also affects cancer metabolism [37, 38]. Additionally, metabolic reprogramming affects the genetic alterations that promote cancer growth and metastasis. To classify MSI and MSS cancers, we first matched *APC* and *TP53* mutations and cancer cell lineages. Using the CATCH model (Fig. 1A), we adjusted the metabolite data with the confounding covariates of metabolic gene expression based on the tensor regression model. Four metabolite features, namely, 3-phosphoglycerate, 6-phosphogluconate, LPE, and GSH, were initially unrelated to MSI cancers and became significantly associated with MSI cancers after adjustment. Metabolic gene expression as a confounder may distort the relationship between metabolite features and MSI cancer status. By controlling the confounding effects, we can determine the actual relevance between metabolites and MSI cancer status.

Plasma metabolites may become promising biomarkers for MSI cancers. There are numerous methods for quantifying MSI, such as immunohistochemistry (IHC) for mismatch repair proteins, PCR analysis of microsatellite markers [4], and NGS [3]. For the diagnosis of MSI cancers, IHC and PCR analyses are frequently utilized; however, for MSI detection, these strategies can only be conducted on cancer tissue samples, not liquid biopsy samples. NGS has been established for the analysis of ctDNA in plasma for MSI identification but has low sensitivity and high cost. A longitudinal study based on targeted metabolomics technology was conducted to validate the findings. The study was approved by the Institutional Review Board (IRB) of the National Cheng Kung University Hospital (NCKUH) (B-ER-110–418). The study is ongoing. Our aim is to develop a series of portfolio-type biomarkers for cancer patients. However, we do not yet have enough samples to perform statistical analysis. Moreover, sarcosine is the most significant adjusted metabolite feature based on the CATCH model for predicting the MSI cancer status. The direct effect was 0.56 (Table 1). Sarcosine was used initially to confirm our results. The preliminary results for 4 MSI cancer patients were obtained. We compared the sarcosine levels of MSI cancer patients with those of healthy controls. There were 1.9- to 3.5-fold increases in sarcosine levels in MSI cancer patients (Additional file 9: Table S6 and Additional file 3: Fig. S3).

Sarcosine (N-methylglycine) is a well-known oncometabolite in prostate cancer. In addition to genetic mutations in MMR (*MLH1*, *MSH2*, *MSH6*, *PMS2*, and *EpCAM*), many mutational features, such as *ACVR2A* and *RNF*, were also present in MSI cancers. Sarcosine may significantly contribute to carcinogenesis via DNA damage and methylation [39]. MSI cancer cells may have increased sarcosine levels owing to MMR genetic mutations. Sarcosine may result in alterations in the cancer genome (Fig. 5). Higher levels of sarcosine in MSI cancers may be one of the possible mechanisms causing genetic alterations in cancer cell lineages that do not carry MMR genetic mutations [24]. Sarcosine and genome alterations can reciprocally regulate each other in MSI cancers (Fig. 5). Through feedback loops, it can become an essential signaling pathway for cancer metabolism.

Many metabolic drugs have been investigated as metabolic therapeutic targets in MSI cancers, such as glycolysis inhibitors (oxamate, lonidamine (LND)) [40, 41], glutamate inhibitors (6-diazo-5-oxo-Lnorleucine, CB839) [42, 43], and lipid metabolism inhibitors (cerulenin, TVB-3664, TOFA) [37]. In our study, dysregulated metabolic pathways resulted in aberrant glycolysis and nucleotide, glutamate, and lipid pathways in MSI cancer cell lines [44]. For example, the synthesis of 3-phosphoglycerate in MSI cancer cells depends on *PFKP*, a critical glycolytic pathway checkpoint. The Warburg effect is facilitated by *PFKP* in malignancies. In previous research, the *HER2*, *EGFR*, AKT-PI3K, and WNT signaling pathways were linked to the regulation of *PFKP*, such as epigenetics in the *PFKP* promoter region or activation of phosphorylation, which were associated with a poor prognosis [45, 46]. Our study established a link between *PFKP* gene expression and 3-phosphoglycerate in MSI cancers with potential clinical applications. We propose that MSI cancer treatment regimens should include the use of glycolysis inhibitors to specifically target *PFKP*.

After adjustment, GSH levels were found to be higher in MSI cancers. There are two distinct facets to GSH metabolism. It has both protective and harmful effects on various cancers. Glutathione overproduction supports cancer cell survival and chemotherapy resistance. The role of GSH in MSI cancer cells should be investigated in future studies. Depletion therapy for glutamine, a precursor of glutathione, or glutamate inhibitors should be evaluated in MSI cancer cells [47].

In Fig. 5, we hypothesized that Hippurate synthetic pathways might be associated with the microbiota and glycine [48]. The metabolite Hippurate is associated with MSS cancers in our model. However, we could not find the biological relevance of Hippurate and associated metabolic genes such as *QDPR*, *FAH*, *PAOX*, *MPST*, and *SLC7A5* in Table 3. In previous studies, Hippurate, a conjugate of glycine and benzoic acid, was used as a metabolomic indicator of gut microbiota diversity [49]. In MSS cancers, metabolism may be affected by environmental factors. Further information must be collected to validate our hypothesis in cancer patients.

One possible therapeutic strategy for MSI cancers is lipid-lowering therapy. Cancer cells utilize lipid metabolism to obtain energy and materials for proliferation, survival, invasion, and metastasis. In the present study, we discovered that MSI cancer cells had elevated levels of lipids, including CE, LPE, and PC. *CHPT1* is also involved in PC and LPE biosynthesis. Clinically, *CHPT1* expression in breast cancer is associated with a poor prognosis [50]. In the future, the *CHPT1*-associated signaling pathway may be targeted in MSI cancer treatment.

## Conclusions

By adjusting the metabolite data with metabolic enzyme genes as confounding covariates, we demonstrated that the CATCH model is an effective tool for predicting MSI cancer status. The adjusted metabolite features offer possible cancer metabolic biomarkers and therapeutic targets for MSI cancers.

## Supplementary Information

**Additional file 1: Fig. S1.** CATCH model for predicting microsatellite instability cancer status.

**Additional file 2: Fig. S2.** CATCH-adjusted versus non-adjusted metabolite data for MSI and MSS cancers.

**Additional file 3: Fig. S3.** Levels of plasma sarcosine in microsatellite instability colorectal and endometrial cancer patients.

**Additional file 4: Table S1.** Cell lines for microsatellite instability and microsatellite stability cancer status.

**Additional file 5: Table S2.** Metabolic genes (87) and the related metabolic pathways.

**Additional file 6: Table S3.** Metabolites (225) involved metabolic pathways.

**Additional file 7: Table S4.** Adjusted metabolite features for microsatellite instability and microsatellite stability cancer status.

**Additional file 8: Table S5.** Relationship between adjusted metabolite features and metabolic genes.

**Additional file 9: Table S6.** Comparison of fold change in sarcosine between microsatellite instability cancer patients and healthy controls.

## Data Availability

Datasets and source codes are available at: https://github.com/H24061024/microsatellite-instability-cancers.The datasets used and analyzed during the current study are available from the corresponding author on reasonable request, and supplementary information files are available for this manuscript.

## Abbreviations

CATCH: Covariate-adjusted tensor classification in high dimensions
CCLE: Cancer cell line encyclopedia
CE: Cholesterol ester
CEA: Carcinoembryonic antigen
CPMG: Carr − purcell − meiboom − gill
CRC: Colorectal cancer
CT: Computed tomography
ctDNA: Circulating tumor DNA
DepMap: Cancer dependency map project
GI: Gastrointestinal
GSH: Reduced glutathione
GU: Genitourinary
GYN: Gynecologic
Hema: Hematologic
HMDB: Human metabolome database
IHC: Immunohistochemistry
KEGG: Kyoto encyclopedia of genes and genomes
LC‒MS: Liquid chromatography‒mass spectrometry
LPE: Lysophosphatidylethanolamine
MMR: Mismatch repair
MSI: Microsatellite instability
MSS: Microsatellite stability
NCKUH: National Cheng Kung university hospital
NGS: Next-generation sequencing
NIH: National institutes of health
NMR: Nuclear magnetic resonance
PC: Phosphatidylcholine
PCR: Polymerase chain reaction
PFKP: Phosphofructokinase 1
QTL: Quantitative trait locus
ROS: Reactive oxygen species
