# Supplementary material for: Controlling the confounding effect of metabolic gene expression to identify actual metabolite targets in microsatellite instability cancers
Source: Hum Genomics. 2023 Mar 6;17:18. doi: 10.1186/s40246-023-00465-9 (PMC9990231; doi:10.1186/s40246-023-00465-9)
Supplement: Supplementary file 1 — Additional file 1: Fig. S1. CATCH model for predicting microsatellite instability cancer status. [file 40246_2023_465_MOESM1_ESM.pdf]

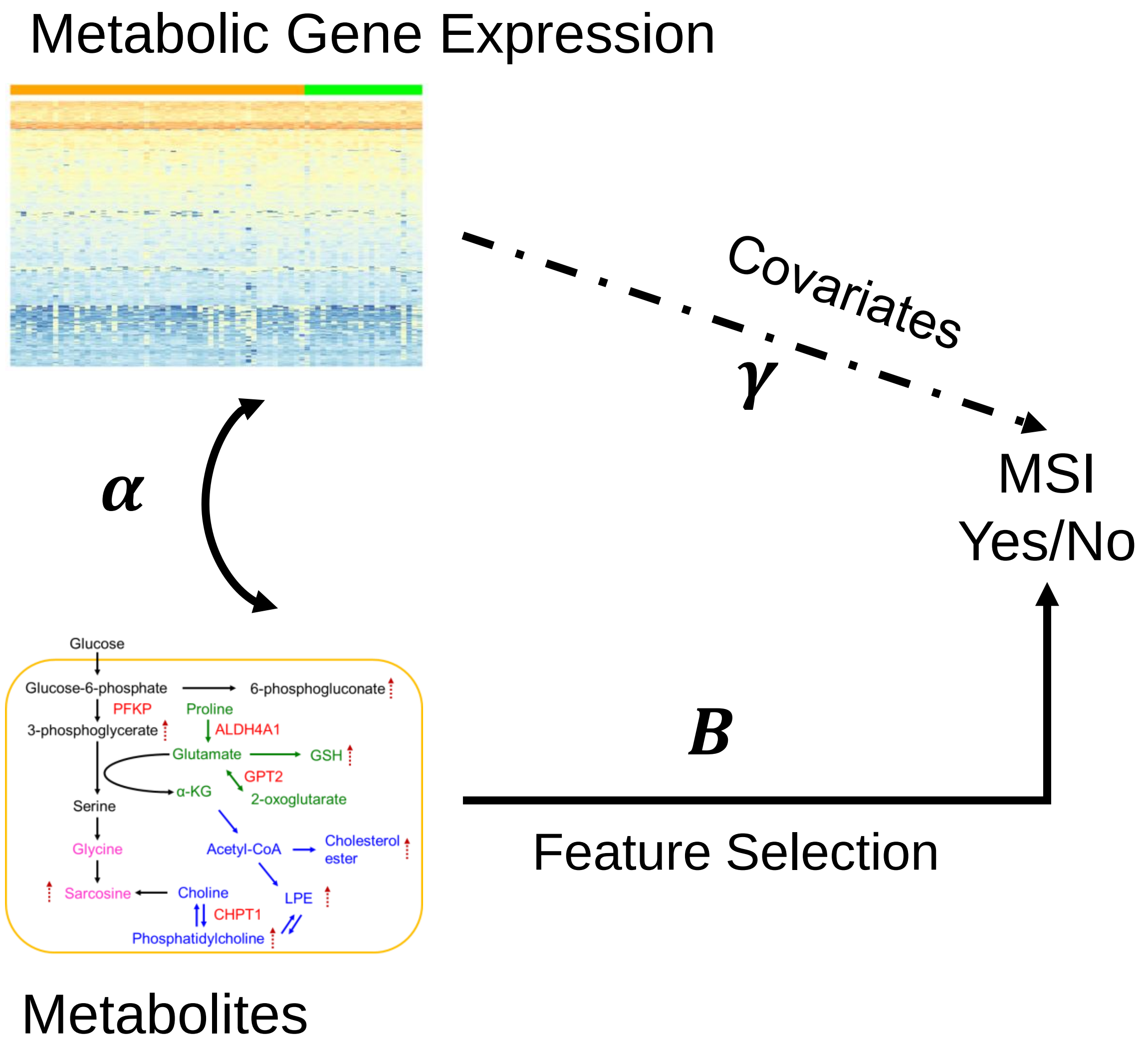

Supplementary Fig. S1. CATCH model for predicting microsatellite instability (MSI) cancer status. The CATCH model can be used to reduce the influence of metabolic gene expression covariates in metabolite data. γ represents the discriminative coefficients for the impact of gene expression on MSI status. α represents the correlation of metabolic gene expression and metabolites. B represents the direct effect of metabolites on MSI status in the tensor discriminant analysis.
