# Supplementary material for: Controlling the confounding effect of metabolic gene expression to identify actual metabolite targets in microsatellite instability cancers
Source: Hum Genomics. 2023 Mar 6;17:18. doi: 10.1186/s40246-023-00465-9 (PMC9990231; doi:10.1186/s40246-023-00465-9)
Supplement: Supplementary file 2 — Additional file 2: Fig. S2. CATCH-adjusted versus non-adjusted metabolite data for MSI and MSS cancers. [file 40246_2023_465_MOESM2_ESM.pdf]

Supplementary Fig. S2. CATCH-adjusted versus non-adjusted metabolite data for MSI and MSS cancers

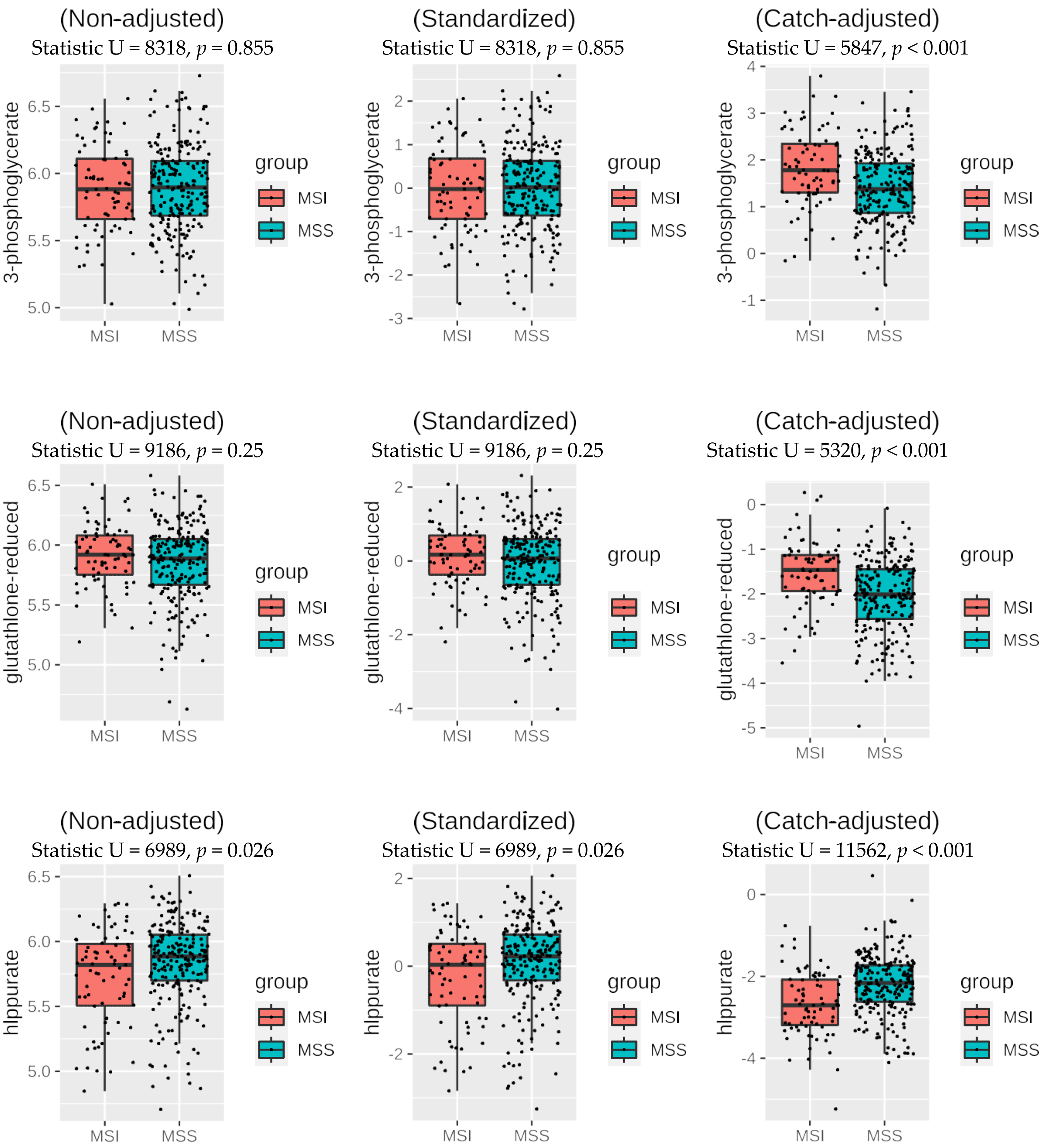

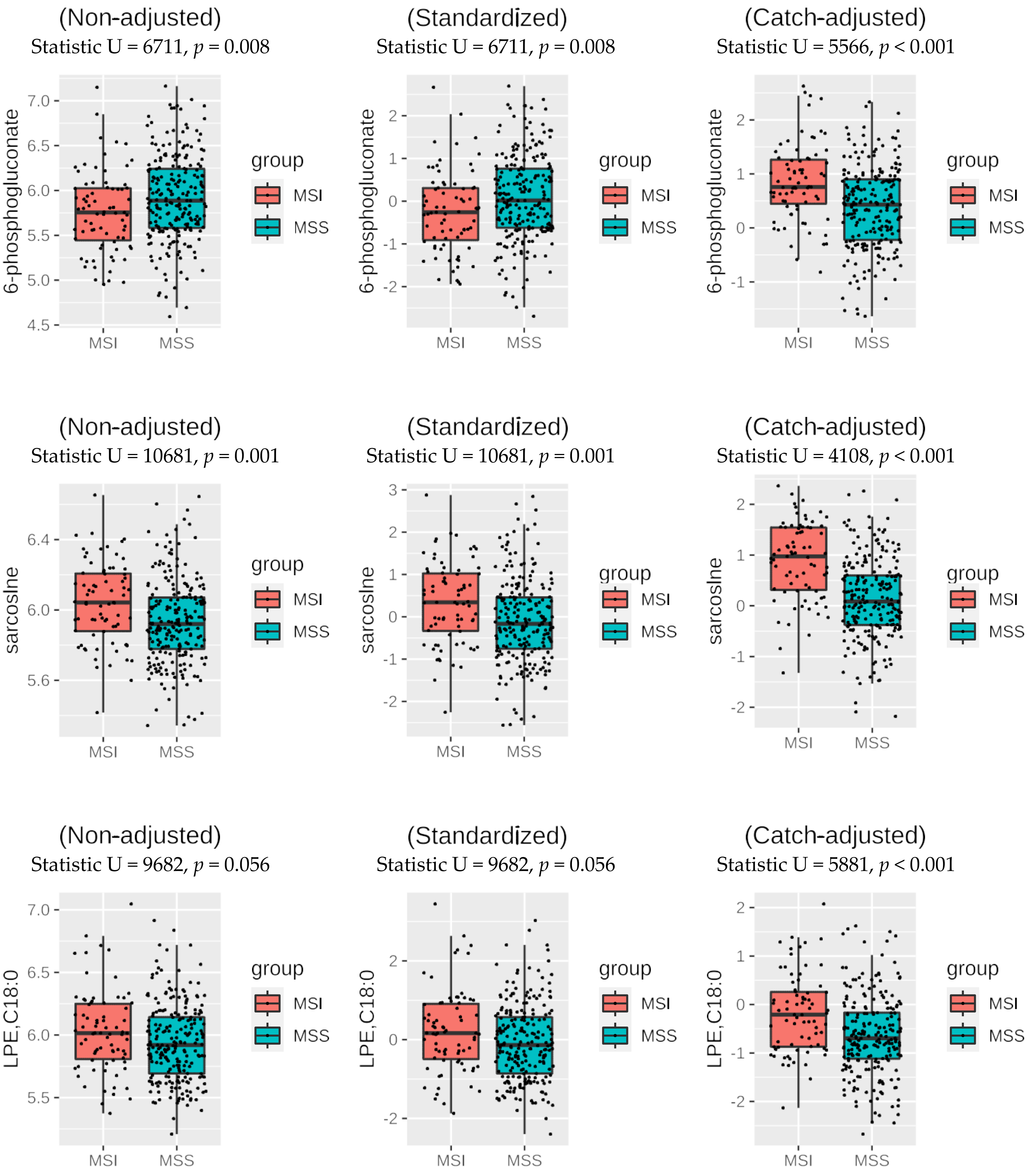
(Non-adjusted)
Statistic U = 6711, p = 0.008
(Standardized)
Statistic U = 6711, p = 0.008
(Catch-adjusted)
Statistic U = 5566, p < 0.001
6-phosphogluconate
group
MSI
MSS
(Non-adjusted)
Statistic U = 10681, p = 0.001
(Standardized)
Statistic U = 10681, p = 0.001
(Catch-adjusted)
Statistic U = 4108, p < 0.001
sarcosine
(Non-adjusted)
Statistic U = 9682, p = 0.056
(Standardized)
Statistic U = 9682, p = 0.056
(Catch-adjusted)
Statistic U = 5881, p < 0.001
LPE,C18:0

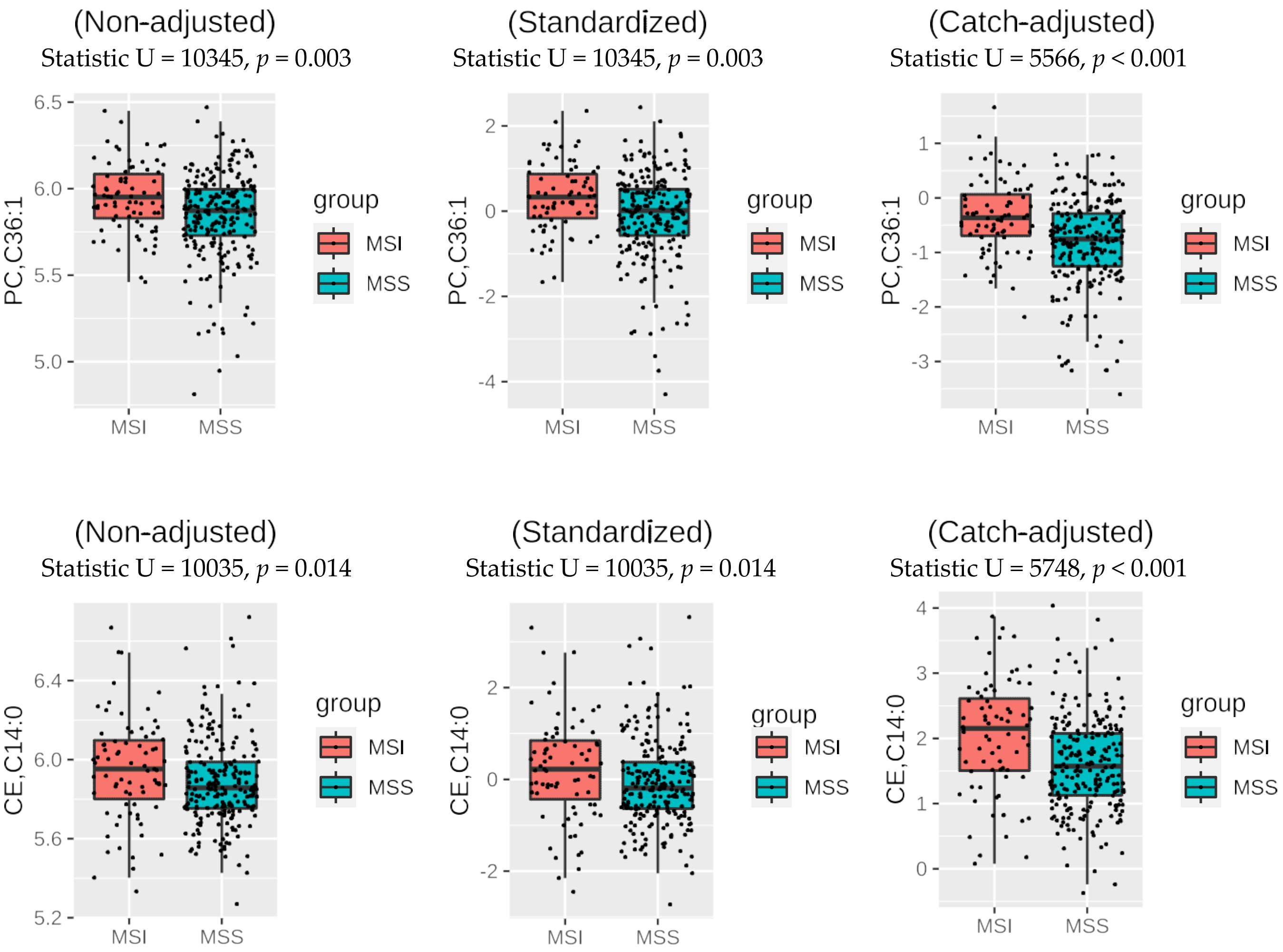
(Non-adjusted)
Statistic U = 10345, p = 0.003
(Standardized)
Statistic U = 10345, p = 0.003
(Catch-adjusted)
Statistic U = 5566, p < 0.001
PC,C36:1
group
MSI
MSS
(Non-adjusted)
Statistic U = 10035, p = 0.014
(Standardized)
Statistic U = 10035, p = 0.014
(Catch-adjusted)
Statistic U = 5748, p < 0.001
CE,C14:0
group
MSI
MSS
