# Supplementary material for: Controlling the confounding effect of metabolic gene expression to identify actual metabolite targets in microsatellite instability cancers
Source: Hum Genomics. 2023 Mar 6;17:18. doi: 10.1186/s40246-023-00465-9 (PMC9990231; doi:10.1186/s40246-023-00465-9)
Supplement: Supplementary file 3 — Additional file 3: Fig. S3. Levels of plasma sarcosine in microsatellite instability colorectal and endometrial cancer patients. [file 40246_2023_465_MOESM3_ESM.pdf]

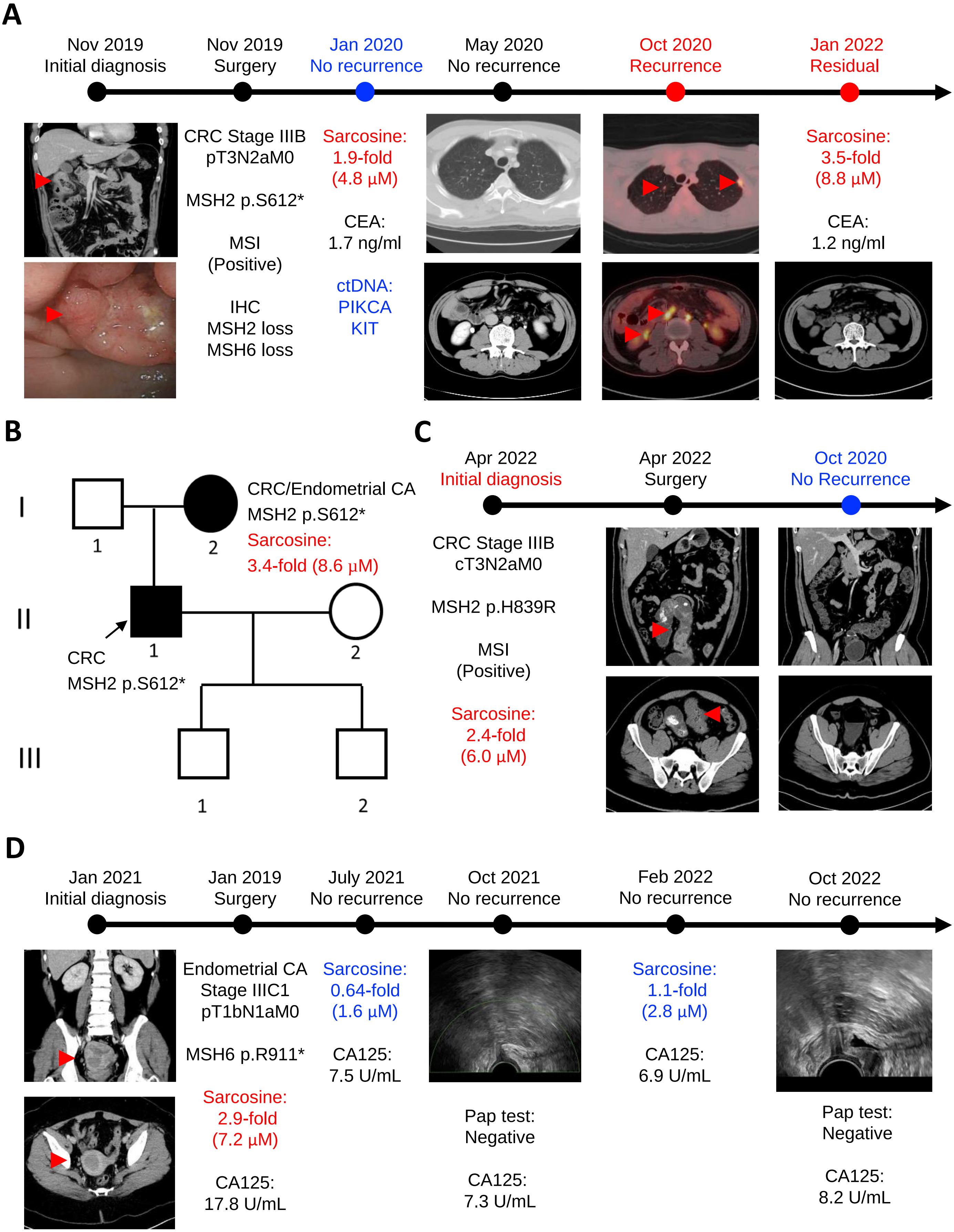
A
Nov 2019
Initial diagnosis
Nov 2019
Surgery
Jan 2020
No recurrence
May 2020
No recurrence
Oct 2020
Recurrence
Jan 2022
Residual
CRC Stage IIIB
pT3N2aM0
MSH2 p.S612*
MSI
(Positive)
IHC
MSH2 loss
MSH6 loss
Sarcosine:
1.9-fold
(4.8 μM)
CEA:
1.7 ng/ml
ctDNA:
PIKCA
KIT
Sarcosine:
3.5-fold
(8.8 μM)
CEA:
1.2 ng/ml
B
I
II
III
1
2
CRC/Endometrial CA
MSH2 p.S612*
Sarcosine:
3.4-fold (8.6 μM)
CRC
MSH2 p.S612*
1
2
1
2
C
Apr 2022
Initial diagnosis
Apr 2022
Surgery
Oct 2020
No Recurrence
CRC Stage IIIB
cT3N2aM0
MSH2 p.H839R
MSI
(Positive)
Sarcosine:
2.4-fold
(6.0 μM)
D
Jan 2021
Initial diagnosis
Jan 2019
Surgery
July 2021
No recurrence
Oct 2021
No recurrence
Feb 2022
No recurrence
Oct 2022
No recurrence
Endometrial CA
Stage IIIC1
pT1bN1aM0
MSH6 p.R911*
Sarcosine:
2.9-fold
(7.2 μM)
CA125:
17.8 U/mL
Sarcosine:
0.64-fold
(1.6 μM)
CA125:
7.5 U/mL
Pap test:
Negative
CA125:
7.3 U/mL
Sarcosine:
1.1-fold
(2.8 μM)
CA125:
6.9 U/mL
Pap test:
Negative
CA125:
8.2 U/mL

Supplementary Fig. S3. Levels of plasma sarcosine in microsatellite instability colorectal and endometrial cancer patients.

In our model, sarcosine strongly correlated with microsatellite instability (MSI) cancers. Sarcosine levels in plasma appear to be a promising monitoring target for cancer recurrence in patients with MSI colorectal cancer (CRC). To validate the findings, we report the preliminary results from four case studies. Metabolite biomarkers, circulating tumor DNA (ctDNA), carcinoembryonic antigen (CEA), CA-125, and image monitoring for cancer recurrence are illustrated in this case study. **A** A 47-year-old man was diagnosed with pathological stage IIIB pT3N2aM0 CRC in November 2019. The patient underwent standard surgical resection followed by adjuvant chemotherapy between December 2019 and August 2020. He carried a germline MSH2 p.S612* genetic mutation. Cancer tissues tested positive for five MSI biomarkers: BAT-25, BAT-26, NR-21, NR-24, and NR-27. Immunohistochemical (IHC) biomarkers have been identified in cancer tissues. In May 2020, six months following surgery, no disease recurrence was detected based on computed tomography (CT) and CEA tests. However, a subsequent positron emission tomography (PET) scan confirmed the recurrence of pulmonary and abdominal metastases in October 2020. Plasma sarcosine levels increased by 1.8-fold (4.8 µM vs. 2.5 µM in healthy controls) in January 2020. Additionally, circulating tumor DNA (ctDNA) with PIK3CA and KIT mutations was isolated. By February 2021, the patient's CEA levels were elevated to twice the normal range (10.1 ng/ml, compared to the normal range of 0-5 ng/ml). The patient received palliative chemotherapy, and a subsequent CT scan showed stable disease in January 2022. Although the CEA level returned to normal, sarcosine levels increased by 3.5-fold (8.8 µM vs. 2.5 µM in the healthy controls). **B** Family pedigree of case 1 and his mother (I-2). From the family pedigree of case 1, we present case 2, a 73-year-old woman who carried a germline MSH2 p.S612* genetic mutation and was diagnosed with CRC and endometrial cancer (EC). She is the mother of case 1. Plasma sarcosine levels increased by 3.4-fold (8.6 µM vs. 2.5 µM in healthy controls). **C** Case 3 is a 28-year-old man who was diagnosed with pathological stage IIIB cT3N2aM0 CRC in April 2022. In this case, the patient underwent surgery followed by adjuvant chemotherapy. There was a germline genetic mutation in MSH2 p.H839R. The five MSI biomarkers tested positive in cancer tissues. Plasma sarcosine levels increased by 2.4-fold (6.0 µM vs. 2.5 µM in healthy controls) before surgery. In October 2022, six months following surgery, no disease recurrence was detected on computed tomography (CT) and carcinoembryonic antigen (CEA) tests**. D** A 49-year-old woman was diagnosed with pathological stage IIIC1 cT1N2aM0 EC in January 2021. She carried the germline MSH6 p.R911* genetic mutation. Sarcosine levels in plasma increased by 2.9-fold (7.2 µM vs. 2.5 µM in healthy controls). By October 2022, no disease recurrence was detected on transvaginal ultrasound for CA-125 (the normal range of 0 to 35 U/mL). Pap smear results were negative. The sarcosine levels also returned to normal limits in July 2021 and February 2022. These results indicated that plasma sarcosine might be a valuable biomarker for early cancer detection, recurrence, and minimal residual disease in MSI cancers, especially in CRC and EC patients.
