# Supplementary material for: Controlling the confounding effect of metabolic gene expression to identify actual metabolite targets in microsatellite instability cancers
Source: Hum Genomics. 2023 Mar 6;17:18. doi: 10.1186/s40246-023-00465-9 (PMC9990231; doi:10.1186/s40246-023-00465-9)
Supplement: Supplementary file 4 — Additional file 4: Table S1. Cell lines for microsatellite instability and microsatellite stability cancer status. [file 40246_2023_465_MOESM4_ESM.pdf]

Supplementary Table S1. Cell lines for MSI and MSS cancer status

Characteristic	MSI (n = 75)	MSS (n = 225)
APC		
Mutation	19(25.3%)	46(20.4%)
Wild type	56(74.7%)	179(79.6%)
TP53		
Mutation	24(32%)	72(32%)
Wild type	51(68%)	153(68%)
Cancer Lineages		
Gastrointestinal	25(33.3%)	75(33.3%)
Breast and GYN	26(34.7%)	78(34.7%)
Hematology	13(17.3%)	39(17.3%)
Genitourinary	5(6.7%)	18(6.7%)
Other	6(8%)	15(8%)

Abbreviations: MSI: microsatellite instability; MSS: microsatellite stability. GYN: Gynecologic cancer
