# Supplementary material for: Controlling the confounding effect of metabolic gene expression to identify actual metabolite targets in microsatellite instability cancers
Source: Hum Genomics. 2023 Mar 6;17:18. doi: 10.1186/s40246-023-00465-9 (PMC9990231; doi:10.1186/s40246-023-00465-9)
Supplement: Supplementary file 5 — Additional file 5: Table S2. Metabolic genes (87) and the related metabolic pathways. [file 40246_2023_465_MOESM5_ESM.pdf]

Supplementary Table S2. Metabolic genes (87) and the related metabolic pathways

Pathway	Metabolism of nucleotides	Metabolism of lipids	Metabolism of amino acids and derivatives	Metabolism of carbohydrates	Total
Number of gene	7	17	32	31	87 Genes
Gene	NME3	CYP51A1	AASS	HS3ST1	
	NME4	AGPS	CHDH	SLC25A13	
	GDA	GALC	MRI1	HEXB	
	NT5C	PLD1	OAT	PFKP	
	DCTD	CERS4	ASNS	PGM1	
	TK2	PTGS1	SMOX	AKR1B1	
	ENTPD3	PLA2G3	PHGDH	XYLB	
		CERK	SLC7A5	XYLT1	
		SQLE	FAH	GALK1	
		SC5D	AADAT	ALDOC	
		CHPT1	HGD	ENO2	
		DHCR24	AGMAT	GNPDA1	
		EPHX2	ASL	B4GALT2	
		SGPP1	MPST	B4GALT6	
		CBR1	IVD	PPP1R3C	
		CERS6	CDO1	CHST3	
		GPX2	GAMT	PFKFB2	
			ASS1	CHPF	
			BHMT2	HS3ST3B1	
			PSAT1	IDUA	
			GLUL	HS6ST1	
			PAOX	HK1	
			QDPR	ST3GAL2	
			ALDH4A1	HK2	
			CBS	GYG1	
			ALDH7A1	CRYL1	
			GPT2	HPSE	
			CKB	PC	
			SLC36A4	CHST2	
			NQO1	B3GNT3	
			PYCR1	SGSH	
			NAT8L		
