# Supplementary material for: Controlling the confounding effect of metabolic gene expression to identify actual metabolite targets in microsatellite instability cancers
Source: Hum Genomics. 2023 Mar 6;17:18. doi: 10.1186/s40246-023-00465-9 (PMC9990231; doi:10.1186/s40246-023-00465-9)
Supplement: Supplementary file 6 — Additional file 6: Table S3. Metabolites (225) involved metabolic pathways. [file 40246_2023_465_MOESM6_ESM.pdf]

Supplementary Table S3. Metabolites (225) involved metabolic pathways

Metabolite	Pathway	Secondary Pathway
**2-aminoadipate**	Metabolism of amino acids and derivatives	Metabolism of lipids
**3-phosphoglycerate**	Metabolism of carbohydrates	
**alpha-glycerophosphate**	Metabolism of carbohydrates	
**4-pyridoxate**	Metabolism of vitamins and cofactors	
**aconitate**	The citric acid (TCA) cycle and respiratory electron transport	
**adenine**	Metabolism of nucleotides	
**adipate**	Metabolism of lipids	
**alpha-ketoglutarate**	Metabolism of carbohydrates	The citric acid (TCA) cycle and respiratory electron transport
**AMP**	Metabolism of nucleotides	
**citrate**	The citric acid (TCA) cycle and respiratory electron transport	
**isocitrate**	The citric acid (TCA) cycle and respiratory electron transport	
**CMP**	Metabolism of nucleotides	
**cystathionine**	Metabolism of amino acids and derivatives	
**cytidine**	Metabolism of nucleotides	
**dCMP**	Metabolism of nucleotides	
**DHAP/glyceraldehyde 3P**	Metabolism of carbohydrates	
**erythrose-4-phosphate**	Metabolism of carbohydrates	
**F1P/F6P/G1P/G6P**	Metabolism of carbohydrates	
**hexoses (HILIC neg)**	Metabolism of carbohydrates	
**fumarate/maleate/alpha-ketoisovalerate**	The citric acid (TCA) cycle and respiratory electron transport	
**glucuronate**	Metabolism of carbohydrates	Biological oxidations
**glutathione oxidized**	Biological oxidations	Metabolism of amino acids and derivatives
**glutathione reduced**	Biological oxidations	Metabolism of amino acids and derivatives
**GMP**	Metabolism of nucleotides	
**guanosine**	Metabolism of nucleotides	
**hippurate**	Metabolism of amino acids and derivatives	
**hypoxanthine**	Metabolism of nucleotides	
**inosine**	Metabolism of nucleotides	
**kynurenine**	Metabolism of carbohydrates	
**lactate**	Metabolism of carbohydrates	
**lactose**	Metabolism of carbohydrates	
**malate**	The citric acid (TCA) cycle and respiratory electron transport	
**NAD**	Metabolism of vitamins and cofactors	
**NADP**	Metabolism of vitamins and cofactors	
**oxalate**	The citric acid (TCA) cycle and respiratory electron transport	
**pantothenate**	Metabolism of vitamins and cofactors	
**PEP**	Metabolism of carbohydrates	
**ribose-5-P/ribulose5-P**	Metabolism of carbohydrates	Biological oxidations
**sorbitol**	Metabolism of carbohydrates	

**succinate/methylmalonate**	The citric acid (TCA) cycle and respiratory electron transport	
**sucrose**	Metabolism of carbohydrates	
**thymine**	Metabolism of nucleotides	
**UMP**	Metabolism of nucleotides	
**UDP-galactose/UDP-glucose**	Metabolism of carbohydrates	Abacavir transport and metabolism/Biological oxidations
**uracil**	Metabolism of nucleotides	Metabolism of amino acids and derivatives
**urate**	Metabolism of nucleotides	
**uridine**	Metabolism of nucleotides	
**xanthine**	Metabolism of nucleotides	
**taurocholate**	Metabolism of lipids	
**glycodeoxycholate/glycochenodeoxycholate**	Metabolism of lipids	
**taurodeoxycholate/taurochenodeoxycholate**	Metabolism of lipids	
**phosphocreatine**	Integration of energy metabolism	
**3-methyladipate/pimelate**	Metabolism of lipids	
**6-phosphogluconate**	Metabolism of carbohydrates	
**alpha-hydroxybutyrate**	Metabolism of lipids	
**2-hydroxyglutarate**	The citric acid (TCA) cycle and respiratory electron transport	
**inositol**	Metabolism of carbohydrates	Inositol phosphate metabolism
**malondialdehyde**	Biological oxidations	Metabolism of lipids
**glycine**	Metabolism of amino acids and derivatives	Metabolism of porphyrins
**alanine**	Metabolism of amino acids and derivatives	
**serine**	Metabolism of amino acids and derivatives	
**threonine**	Metabolism of amino acids and derivatives	
**methionine**	Metabolism of amino acids and derivatives	
**aspartate**	Metabolism of amino acids and derivatives	
**glutamate**	Metabolism of amino acids and derivatives	
**asparagine**	Metabolism of amino acids and derivatives	
**glutamine**	Metabolism of amino acids and derivatives	
**histidine**	Metabolism of amino acids and derivatives	
**arginine**	Metabolism of amino acids and derivatives	Metabolism of nitric oxide
**lysine**	Metabolism of amino acids and derivatives	
**valine**	Metabolism of amino acids and derivatives	
**leucine**	Metabolism of amino acids and derivatives	
**isoleucine**	Metabolism of amino acids and derivatives	
**phenylalanine**	Metabolism of amino acids and derivatives	
**tyrosine**	Metabolism of amino acids and derivatives	
**tryptophan**	Metabolism of amino acids and derivatives	
**proline**	Metabolism of amino acids and derivatives	
**cis/trans-hydroxyproline**	Metabolism of amino acids and derivatives	
**ornithine**	Metabolism of amino acids and derivatives	
**citrulline**	Metabolism of amino acids and derivatives	

**taurine**	Metabolism of amino acids and derivatives	
**5-HIAA**	Metabolism of amino acids and derivatives	
**serotonin**	Metabolism of amino acids and derivatives	
**GABA**	Metabolism of amino acids and derivatives	
**acetylglycine**	Metabolism of amino acids and derivatives	
**dimethylglycine**	Metabolism of amino acids and derivatives	
**homocysteine**	Metabolism of amino acids and derivatives	
**SDMA/ADMA**	Metabolism of amino acids and derivatives	
**NMMA**	Metabolism of amino acids and derivatives	
**allantoin**	Metabolism of amino acids and derivatives	
**anthranilic acid**	Metabolism of amino acids and derivatives	
**kynurenic acid**	Metabolism of amino acids and derivatives	
**5-adenosylhomocysteine**	Metabolism of amino acids and derivatives	
**carnosine**	Metabolism of amino acids and derivatives	
**N-carbamoyl-beta-alanine**	Metabolism of amino acids and derivatives	
**thiamine**	Metabolism of vitamins and cofactors	
**niacinamide**	Metabolism of vitamins and cofactors	
**betaine**	Metabolism of amino acids and derivatives	
**choline**	Metabolism of lipids	
**alpha-glycerophosphocholine**	Metabolism of lipids	
**acetylcholine**	Metabolism of lipids	
**creatine**	Metabolism of amino acids and derivatives	Integration of energy metabolism
**creatinine**	Metabolism of amino acids and derivatives	
**thyroxine**	Metabolism of amino acids and derivatives	Integration of energy metabolism
**trimethylamine-N-oxide**	Metabolism of lipids	Metabolism of amino acids and derivatives
**hexoses (HILIC pos)**	Metabolism of carbohydrates	
**adenosine**	Metabolism of nucleotides	
**thymidine**	Metabolism of nucleotides	
**xanthosine**	Metabolism of nucleotides	
**2-deoxyadenosine**	Metabolism of nucleotides	
**2-deoxycytidine**	Metabolism of nucleotides	
**cAMP**	Metabolism of nucleotides	
**cotinine**	尼古丁代謝	
**pipecolic acid**	Metabolism of amino acids and derivatives	
**pyroglutamic acid**	Metabolism of amino acids and derivatives	
**1-methylnicotinamide**	Metabolism of amino acids and derivatives	
**butyrobetaine**	Metabolism of amino acids and derivatives	
**putrescine**	Metabolism of amino acids and derivatives	
**methionine sulfoxide**	Biological oxidations	
**carnitine**	Metabolism of lipids	
**acetylcarnitine**	Metabolism of lipids	

**propionylcarnitine**	Metabolism of lipids
**malonylcarnitine**	Metabolism of lipids
**butyrylcarnitine/isobutyrylcarnitine**	Metabolism of lipids
**valerylcarnitine/isovalerylcarnitine/2-methyl**	Metabolism of lipids
**hexanoylcarnitine**	Metabolism of lipids
**heptanoylcarnitine**	Metabolism of lipids
**lauroylcarnitine**	Metabolism of lipids
**myristoylcarnitine**	Metabolism of lipids
**palmitoylcarnitine**	Metabolism of lipids
**stearoylcarnitine**	Metabolism of lipids
**oleylcarnitine**	Metabolism of lipids
**arachidonyl_carnitine**	Metabolism of lipids
**sarcosine**	Metabolism of amino acids and derivatives
**beta-alanine**	Metabolism of amino acids and derivatives
**anserine**	Metabolism of amino acids and derivatives
**C14:0 LPC**	Metabolism of lipids
**C16:1 LPC**	Metabolism of lipids
**C16:0 LPC**	Metabolism of lipids
**C18:2 LPC**	Metabolism of lipids
**C18:1 LPC**	Metabolism of lipids
**C18:0 LPC**	Metabolism of lipids
**C20:4 LPC**	Metabolism of lipids
**C20:3 LPC**	Metabolism of lipids
**C22:6 LPC**	Metabolism of lipids
**C16:0 LPE**	Metabolism of lipids
**C18:1 LPE**	Metabolism of lipids
**C18:0 LPE**	Metabolism of lipids
**C20:4 LPE**	Metabolism of lipids
**C22:6 LPE**	Metabolism of lipids
**C32:2 PC**	Metabolism of lipids
**C32:1 PC**	Metabolism of lipids
**C32:0 PC**	Metabolism of lipids
**C34:4 PC**	Metabolism of lipids
**C34:3 PC**	Metabolism of lipids
**C34:2 PC**	Metabolism of lipids
**C34:1 PC**	Metabolism of lipids
**C36:4 PC-A**	Metabolism of lipids
**C36:4 PC-B**	Metabolism of lipids
**C36:3 PC**	Metabolism of lipids
**C36:2 PC**	Metabolism of lipids
**C36:1 PC**	Metabolism of lipids

**C38:6 PC**	Metabolism of lipids
**C38:5 PC**	Metabolism of lipids
**C38:4 PC**	Metabolism of lipids
**C38:2 PC**	Metabolism of lipids
**C40:6 PC**	Metabolism of lipids
**C14:0 SM**	Metabolism of lipids
**C16:1 SM**	Metabolism of lipids
**C16:0 SM**	Metabolism of lipids
**C18:2 SM**	Metabolism of lipids
**C18:1 SM**	Metabolism of lipids
**C18:0 SM**	Metabolism of lipids
**C22:1 SM**	Metabolism of lipids
**C22:0 SM**	Metabolism of lipids
**C24:1 SM**	Metabolism of lipids
**C24:0 SM**	Metabolism of lipids
**C34:2 DAG**	Metabolism of lipids
**C34:1 DAG**	Metabolism of lipids
**C36:2 DAG**	Metabolism of lipids
**C36:1 DAG**	Metabolism of lipids
**C14:0 CE**	Metabolism of lipids
**C16:1 CE**	Metabolism of lipids
**C16:0 CE**	Metabolism of lipids
**C18:3 CE**	Metabolism of lipids
**C18:2 CE**	Metabolism of lipids
**C18:1 CE**	Metabolism of lipids
**C18:0 CE**	Metabolism of lipids
**C20:5 CE**	Metabolism of lipids
**C20:4 CE**	Metabolism of lipids
**C20:3 CE**	Metabolism of lipids
**C22:6 CE**	Metabolism of lipids
**C46:2 TAG**	Metabolism of lipids
**C46:1 TAG**	Metabolism of lipids
**C46:0 TAG**	Metabolism of lipids
**C48:3 TAG**	Metabolism of lipids
**C48:2 TAG**	Metabolism of lipids
**C48:1 TAG**	Metabolism of lipids
**C48:0 TAG**	Metabolism of lipids
**C50:3 TAG**	Metabolism of lipids
**C50:2 TAG**	Metabolism of lipids
**C50:1 TAG**	Metabolism of lipids
**C50:0 TAG**	Metabolism of lipids

**C52:5 TAG**	Metabolism of lipids
**C52:4 TAG**	Metabolism of lipids
**C52:3 TAG**	Metabolism of lipids
**C52:2 TAG**	Metabolism of lipids
**C52:1 TAG**	Metabolism of lipids
**C54:7 TAG**	Metabolism of lipids
**C54:6 TAG**	Metabolism of lipids
**C54:5 TAG**	Metabolism of lipids
**C54:4 TAG**	Metabolism of lipids
**C54:3 TAG**	Metabolism of lipids
**C54:2 TAG**	Metabolism of lipids
**C54:1 TAG**	Metabolism of lipids
**C56:8 TAG**	Metabolism of lipids
**C56:7 TAG**	Metabolism of lipids
**C56:6 TAG**	Metabolism of lipids
**C56:5 TAG**	Metabolism of lipids
**C56:4 TAG**	Metabolism of lipids
**C56:3 TAG**	Metabolism of lipids
**C56:2 TAG**	Metabolism of lipids
**C58:8 TAG**	Metabolism of lipids
**C58:7 TAG**	Metabolism of lipids
**C58:6 TAG**	Metabolism of lipids

SDMA/ADMA: symmetric dimethylarginine/asymmetric dimethylarginine
NMMA: N-monomethyl-arginine
LPC: lysophosphatidylcholine
PC: phosphatidylcholine
LPE: lysophosphatidylethanolamine
CE: cholesterol ester
SM: sphingomyelin
TAG: triacylglycerol
DAG: diacylglycerol
