# Supplementary material for: Controlling the confounding effect of metabolic gene expression to identify actual metabolite targets in microsatellite instability cancers
Source: Hum Genomics. 2023 Mar 6;17:18. doi: 10.1186/s40246-023-00465-9 (PMC9990231; doi:10.1186/s40246-023-00465-9)
Supplement: Supplementary file 7 — Additional file 7: Table S4. Adjusted metabolite features for microsatellite instability and microsatellite stability cancer status. [file 40246_2023_465_MOESM7_ESM.pdf]

Supplementary Table S4. Adjusted metabolite features for MSI and MSS cancer status

depMapID	y_ref.	Lineage	APC	TP53	Hippurate	3-phosphoglycerate	C14:0 CE	C18:0 LPE	6-phosphogluconate	C36:1 PC	Glutathione reduced	Sarcosine
ACH-000001	MSS	Breast and GYN	M	M	1.288529417	-0.090844362	0.985707045	-0.349450347	0.229870915	-1.941253372	-0.958505594	-0.15730935
ACH-000006	MSS	Hema	M	M	1.015307757	-0.926014647	0.351737573	2.170698703	-1.838605995	-1.236698549	-1.891622802	-0.31329414
ACH-000007	MSS	GI	M	M	0.736246645	-1.59754796	0.483454324	-0.752291426	-1.379009092	0.798653342	-0.670195727	-1.06866556
ACH-000009	MSS	GI	W	W	1.175339539	-0.365907566	-1.490701492	-0.701220679	-1.100661949	0.186391429	0.017511272	-0.0561482
ACH-000013	MSS	Breast and GYN	M	M	0.020671971	0.556203646	0.405660121	-0.419802799	0.744772211	-1.357173105	0.285191625	0.533616939
ACH-000015	MSS	other	W	W	0.590136618	-0.105938646	-1.183853429	-0.600352295	-1.904468891	-1.172610107	0.308789405	0.617130754
ACH-000017	MSS	Breast and GYN	M	M	1.918514629	-1.039665718	0.213667285	-1.498330998	-0.025312508	-0.863087943	-2.47093689	-1.20232476
ACH-000018	MSS	GU	M	W	0.325716302	-0.033046992	0.621400065	0.420883313	0.522536661	-0.846608985	0.258472812	0.884692918
ACH-000022	MSS	GI	M	M	-0.70237291	-1.189482101	0.772194194	-0.858877506	-1.535616061	-0.042598149	0.300441599	-0.40852313
ACH-000024	MSS	Hema	M	M	-0.00363645	0.168362202	-1.034098822	-1.770007894	0.329577425	-1.993311093	1.197418876	0.123459006
ACH-000026	MSS	GU	M	M	-0.56288825	0.029046685	-0.475937813	1.400460349	1.47721653	0.410040731	1.203409705	1.3882273
ACH-000028	MSS	Breast and GYN	M	M	0.430595187	2.437901262	-1.223737344	-0.603101426	0.699166456	-3.551051212	1.259872047	0.342893329
ACH-000042	MSS	GI	M	M	1.04767651	0.442752882	-2.479336005	-2.29239057	-1.362886309	-0.6201597	-1.458188277	-0.96619148
ACH-000048	MSS	Breast and GYN	M	M	1.187714402	-0.270147522	-0.607553905	-1.60533812	-0.074400649	-0.807690349	-0.951433928	-0.39693434
ACH-000052	MSS	other	M	W	1.256033595	-1.119179536	-0.341180491	-1.250017155	0.769097765	-0.539144339	-1.15137583	-0.59433258
ACH-000056	MSS	Hema	M	M	0.072091331	0.412476369	0.422886103	-0.322806962	0.275203023	-0.716655681	0.786115405	0.889947214
ACH-000062	MSS	other	M	W	-0.39555813	0.35866752	0.612302553	0.425377041	-0.051838954	-0.158307129	1.426101238	-0.84482247
ACH-000068	MSS	Hema	M	W	-0.36212545	-0.256113487	-0.997286989	-0.086897919	-2.077632091	-0.235292792	1.741205845	-1.24911747
ACH-000073	MSS	Hema	M	M	-1.86475487	1.987992039	0.547048443	-0.203472029	1.220630421	0.138192446	0.815739026	-2.95688956
ACH-000076	MSS	Hema	M	M	-0.40005599	-2.395960571	-0.52893502	-0.482548821	-0.307110127	1.329559316	-0.022653167	-0.13364959
ACH-000084	MSS	Hema	M	M	-1.28329935	-0.108022658	0.575939429	1.091282275	1.755763245	-0.097703955	-1.785546794	1.480300994
ACH-000091	MSS	Breast and GYN	M	W	0.072117779	-0.631344584	-0.125681667	1.237793366	0.166091849	-0.380075528	-0.190245766	0.092222336
ACH-000097	MSS	Breast and GYN	M	M	-0.05062236	0.929097204	-0.511249203	-0.974191815	1.064864089	-0.494160928	1.018361544	1.688226747
ACH-000101	MSS	Hema	M	W	0.002235424	-1.300148841	-0.268923593	-0.363755337	-1.629054428	-0.95459177	0.630084587	-1.65158084
ACH-000109	MSS	other	M	W	-0.07987713	0.816153083	-0.997665154	-1.479885815	1.070310928	-1.443552264	-0.824777012	0.607235575
ACH-000111	MSS	Breast and GYN	M	M	-0.4702434	0.438220733	1.861063124	-0.071764065	0.626894649	-0.042162086	-1.492799803	-0.16109984
ACH-000116	MSS	Breast and GYN	M	W	-1.36590652	-0.313390246	-1.339808499	-0.138611051	-0.087266736	-2.901720507	-0.190233409	-0.03686445
ACH-000117	MSS	Breast and GYN	M	W	0.958743122	-0.507652527	0.063338921	-0.97604173	2.260779079	-1.007128319	-0.815332327	-0.07549913
ACH-000121	MSS	other	M	M	0.83023433	-0.90469762	-0.63823486	0.642425444	-0.979700949	0.134100598	-0.100178005	-0.53717073
ACH-000132	MSS	Breast and GYN	M	W	0.999510164	-0.574773567	-1.001955364	-1.455965739	-1.030198392	-0.444131603	-1.028751352	-1.1052298
ACH-000140	MSS	Hema	M	M	1.235560821	-1.034201294	0.636758466	0.766381967	0.53789879	1.045589497	-3.697737709	0.751737271
ACH-000141	MSS	GI	M	M	-0.64398579	0.00191145	-1.122235118	-0.046697094	0.519276997	0.822674269	0.849409309	-0.30196614
ACH-000146	MSS	Hema	M	W	-0.48202856	-0.104804468	0.265857192	1.39803699	1.674597054	-1.392190238	1.077547903	-0.48726867
ACH-000147	MSS	Breast and GYN	M	M	-0.77745415	0.485237706	1.669964364	0.530968765	-1.158093665	0.208983096	0.119862455	0.632132152
ACH-000150	MSS	other	W	M	-0.52557211	-0.729195525	-0.480623177	-0.992010499	-2.313113262	0.131254511	0.992825628	-0.73837432
ACH-000156	MSS	Hema	M	M	0.194504295	0.870676463	-0.575827899	-1.230148864	1.068169058	0.279634516	-0.864224617	-1.20012565
ACH-000158	MSS	Hema	M	M	0.322457974	-0.289191941	-0.615277433	-0.231154208	-0.979881216	-0.263853305	1.28634006	0.121511932
ACH-000168	MSS	Hema	M	W	-0.16258954	-0.908781498	0.149401725	-0.045965887	-2.322965383	-0.305054381	0.795145231	-1.34231882
ACH-000173	MSS	Breast and GYN	M	W	0.161523174	-0.327671346	-2.111717526	-0.37161825	0.061423526	0.368999747	0.592866359	-0.00962655

ACH-000181	MSS	GI	M	M	0.346384422	0.70668247	0.203833378	-0.610237745	0.887137607	-0.144878333	-0.285932895	-0.52758736
ACH-000182	MSS	GI	M	M	0.127072985	1.404356457	1.661203516	0.829161292	-1.063468323	0.213265322	0.281111912	0.331473068
ACH-000183	MSS	Hema	M	W	-2.03692832	-1.173514466	0.467171722	-0.335314576	0.800269015	1.416355632	-0.141824185	-1.08541621
ACH-000192	MSS	Breast and GYN	M	W	2.182385887	0.336347514	-0.739539586	-0.904611639	-1.293161203	-0.051385598	-0.186779903	1.063031199
ACH-000193	MSS	Hema	M	M	0.686983048	0.775147558	-0.317470905	1.537167611	0.389416075	1.851314442	1.226856258	-0.06884439
ACH-000194	MSS	other	M	M	0.504223404	-1.79904838	-1.148801393	0.416074618	-0.968554672	0.452064654	-1.028158367	-0.24117693
ACH-000196	MSS	Breast and GYN	M	W	-0.50716553	-0.453500171	-1.524047925	-0.279346134	0.540900248	-0.309327184	1.268569285	-0.85191208
ACH-000197	MSS	Hema	M	M	1.683659117	0.421932711	-0.927074663	-0.455165862	-1.154635326	0.125557579	-0.221087287	0.658306911
ACH-000201	MSS	other	M	M	0.667332691	-1.296303777	2.309923756	-0.506281819	0.67816001	0.54446963	-1.351854517	1.60526978
ACH-000202	MSS	GI	W	M	-2.15068348	-0.420220719	-0.029659518	0.95118929	-0.598199575	1.167190626	-0.105113563	-1.80522229
ACH-000204	MSS	Hema	M	M	-0.24554809	0.904069208	-0.604051001	-0.388039837	1.343992287	-1.049252747	0.419267169	0.699526192
ACH-000205	MSS	GI	M	M	-0.44681622	0.767891331	-0.018179685	1.237599904	-0.064282922	0.523461725	0.584735149	-0.35129231
ACH-000212	MSS	Breast and GYN	M	W	0.261416697	-0.499465784	0.034946249	0.886033552	-0.437564135	0.797677574	-0.432704685	-1.41209232
ACH-000213	MSS	GI	M	M	0.957388482	-1.926162437	-1.754304424	-1.327237251	-1.363747579	-0.814235952	-2.279124584	-0.40614376
ACH-000218	MSS	Hema	M	M	3.612978636	0.631321256	-0.398232194	-1.156275882	-0.032272514	-0.809333753	0.334898736	-0.31772182
ACH-000223	MSS	Breast and GYN	M	W	0.956940843	0.201340546	-1.106902121	1.287509046	0.52668997	-3.018818546	-2.275320057	0.953431966
ACH-000225	MSS	GI	M	M	0.692933561	-0.686869291	1.123820018	-0.366224819	0.157265364	-0.076666628	-1.303549884	-0.94036481
ACH-000228	MSS	GI	M	M	0.819025143	-0.525332804	0.962103416	0.037078811	-1.533127727	0.56691618	-0.347318627	-0.11838453
ACH-000235	MSS	GI	W	M	-1.50353075	1.500960115	0.739237777	0.057672511	0.018638656	0.311579218	0.519669241	1.383507811
ACH-000236	MSS	GI	W	W	0.837655706	-0.801153091	0.149155214	-1.426275421	-1.864028448	0.792564471	-1.246376976	0.20318545
ACH-000237	MSS	Breast and GYN	M	W	-0.48094547	-0.372787398	-0.673101266	-0.635581706	-0.373152607	0.542581453	0.539626843	-1.11089675
ACH-000239	MSS	GI	W	M	-2.33943983	-1.094966376	0.38699642	1.343046805	0.508394796	1.830825828	-0.898631534	1.137157598
ACH-000242	MSS	GU	M	M	-2.04935988	0.910569608	-0.253115155	1.076206713	1.18089856	-1.123946439	2.232372327	1.268197986
ACH-000248	MSS	Breast and GYN	M	M	0.799563171	-1.068506291	-0.121672794	-0.720457653	1.23694001	0.39604006	-1.353278525	-0.83486646
ACH-000249	MSS	GI	W	M	-0.78814913	-1.964352284	-0.289060862	0.02073077	-0.058496237	-0.443179726	-0.065390157	0.200258669
ACH-000252	MSS	GI	M	M	-1.13360827	-1.151206049	0.819395567	-1.294633675	-0.6521041	0.095511074	-1.775807568	-2.11534069
ACH-000253	MSS	GI	W	M	-1.55440139	-3.353407108	-0.353721956	0.094841841	-1.684721239	1.392514638	0.400459933	-0.488543
ACH-000258	MSS	Breast and GYN	W	M	-1.53446655	-0.863772318	-0.276864951	0.404408027	-1.061888222	0.447655298	0.643846645	-1.38339439
ACH-000266	MSS	GI	M	M	-0.14822102	2.141716098	0.102357804	-0.268326745	1.061612664	-0.480119428	1.048175674	0.13842737
ACH-000268	MSS	GI	M	M	-1.87076771	-0.043567785	0.063656669	-0.775293328	0.587823359	-1.100377659	-0.469417462	-1.1133877
ACH-000270	MSS	GI	M	W	-0.75000809	0.119901984	1.237269717	2.191476088	-0.150812691	0.003475944	0.408604752	-0.21203596
ACH-000271	MSS	Hema	M	W	0.24354863	-0.815141877	-1.231364134	2.491898873	-1.41836366	0.112762269	0.568679408	-1.37523302
ACH-000276	MSS	Breast and GYN	M	M	-0.24227627	-0.027772432	-1.023011568	0.148507885	-1.649550232	0.220567246	-0.57102098	0.793235007
ACH-000277	MSS	Breast and GYN	M	W	-1.36434482	1.359872476	2.689341317	1.212958633	0.699424358	-0.642551089	1.402170199	0.753248786
ACH-000278	MSS	Breast and GYN	M	M	0.510471325	-2.263918873	0.110412237	-0.414099313	-0.211742487	0.579796466	1.013207625	0.204173954
ACH-000280	MSS	Breast and GYN	M	M	0.823584646	-1.382195067	0.497192875	0.068493755	-0.081530052	0.105953772	0.380172839	-1.55192388
ACH-000286	MSS	GI	W	M	-0.09049259	0.286718623	-0.070369973	-0.704292348	1.323541679	-0.087671936	-0.597714817	1.329674027
ACH-000288	MSS	Breast and GYN	M	M	2.137439279	-1.409552492	-0.647521648	-2.10753881	-1.322796627	-2.460511534	-1.454985676	0.532318212
ACH-000291	MSS	Breast and GYN	M	M	0.183155371	-0.265164654	0.409377068	-0.390026712	1.03149556	-1.790438697	-0.494686735	-0.00526197
ACH-000293	MSS	Breast and GYN	M	M	1.484051513	-1.642001929	0.120285104	-0.706613758	-0.212756008	0.786359664	-0.930584397	0.306976486
ACH-000296	MSS	GI	W	M	0.613418637	-0.046898807	-1.695380639	0.67088473	-0.47009942	-3.009293782	-0.163420631	-0.82091692

ACH-000302	MSS	Breast and GYN	M	M	-1.06393821	0.680099607	-0.133706337	1.239293565	-0.08077556	0.20295264	-0.11318106	0.499926309
ACH-000308	MSS	Breast and GYN	M	M	0.205051199	0.200259066	-1.154844002	-0.961508456	0.113834854	-0.230489296	0.649090213	0.427366828
ACH-000315	MSS	Hema	M	W	0.535357705	0.046219836	-0.358396025	-1.227338771	-0.619261669	0.946139229	1.438338391	0.293672111
ACH-000320	MSS	GI	M	M	0.696752807	-2.022421374	2.960392952	0.588742768	0.741832143	0.731071045	-1.933461689	-0.28280576
ACH-000324	MSS	Breast and GYN	M	M	1.317690923	0.252706875	-0.907066626	2.42351403	0.4696442	0.383443992	-0.244844809	-0.72772504
ACH-000326	MSS	Hema	M	W	-0.08989977	-0.171104686	-2.223872704	-0.416900548	-1.11783455	1.196474145	0.567918183	-1.22622998
ACH-000330	MSS	Breast and GYN	M	M	0.12315968	-0.61980233	-1.586154763	-1.538265716	-0.197346271	-0.334230265	-2.172303072	-0.010171
ACH-000333	MSS	Breast and GYN	M	M	1.195202596	-0.783525827	0.627958839	-0.197677367	-0.572079975	-0.185935773	0.09321614	-0.63191854
ACH-000342	MSS	GI	W	M	0.593713656	-0.751082492	0.318927021	-1.299457408	0.15941324	-2.709535006	-1.359850122	-0.82821399
ACH-000343	MSS	other	M	W	-0.027799	0.949415972	0.184324626	-0.317757255	-1.782915539	-1.399672789	0.15878862	-0.73854494
ACH-000347	MSS	GI	M	W	0.56754638	0.595445096	-0.688010839	-0.594412887	0.074411225	0.736051109	0.225165726	2.20110067
ACH-000349	MSS	Breast and GYN	M	M	0.586307471	0.011729164	-0.134506972	0.388009327	-0.649576295	0.254707635	1.085770927	-0.42637438
ACH-000350	MSS	GI	W	M	0.757291072	-0.460519119	-0.051653749	-0.413710567	-1.154625995	0.094611707	0.192047785	-0.28504339
ACH-000353	MSS	GI	M	W	-0.74288849	-0.534936798	0.261386746	0.121978371	1.518843921	0.280861378	-0.191332026	1.083081302
ACH-000357	MSS	Hema	M	W	-0.17887795	0.417261316	0.071267247	-1.026842304	-0.137244682	-0.416882958	-0.519007581	-0.22235155
ACH-000360	MSS	GI	M	M	-2.04375082	1.927797904	-0.936781588	1.667991932	-0.235052423	-0.071854262	-0.050967316	0.294626553
ACH-000369	MSS	Hema	M	M	1.900904925	-0.941255833	1.158265381	-2.566525955	0.676187741	0.11697035	-0.184556031	-0.01763612
ACH-000381	MSS	GI	W	W	-0.47400159	-0.201263989	-0.93111324	-1.426995124	1.34934892	-2.854114979	1.606247404	-0.37791222
ACH-000382	MSS	other	W	M	-0.27232867	0.59499492	0.018141556	0.800962436	0.468959662	-0.363980881	-1.407505969	0.032953941
ACH-000384	MSS	GU	M	M	0.876894864	-0.322046405	1.108505272	0.672143309	-0.61370165	-0.06014026	-0.208658093	1.122890796
ACH-000386	MSS	Hema	M	W	0.068124062	1.585484267	-0.680109035	-0.421921175	0.39517414	-0.345348748	0.158304695	0.744007188
ACH-000387	MSS	Hema	M	W	0.280029895	0.254863243	0.3267464	0.15331573	-1.169764702	0.225674275	0.782981032	0.172223386
ACH-000397	MSS	Breast and GYN	M	W	0.523388531	-0.991571017	0.081029974	-0.501382986	-0.438292332	-0.633820631	-1.942249799	-2.19472198
ACH-000400	MSS	GI	W	M	0.071095028	-1.508794679	-0.359568331	-1.89152288	-0.774765009	-0.096709585	-1.111894654	-0.7137588
ACH-000403	MSS	GI	W	M	1.659436376	-0.839426868	-0.096490077	-0.92898903	-0.734672222	0.679655765	-1.572444215	-0.3915188
ACH-000406	MSS	Hema	M	W	-0.19300883	-0.237975135	-0.19949331	-0.645665673	0.488368161	-0.663891502	0.556606225	-0.00088234
ACH-000407	MSS	Breast and GYN	M	W	0.362730451	0.252861383	-0.659400062	-0.288549533	0.907808792	-0.970174691	0.041744811	0.293807642
ACH-000409	MSS	Breast and GYN	M	W	0.228069542	-0.067268123	1.540498163	0.572614483	-0.205103087	0.339519702	-0.714686339	-0.6444365
ACH-000417	MSS	GI	M	M	0.633637317	0.971599023	-0.718010029	-0.245243418	1.475140806	1.309892986	0.668765682	-0.40119351
ACH-000421	MSS	GI	W	M	-0.80810641	-0.260850308	-0.461455744	0.668387014	0.639269991	0.995525528	-1.113075869	-0.97968777
ACH-000427	MSS	GI	M	M	0.047683238	0.774508187	1.216225942	-1.253002614	-0.378895595	0.754633679	0.394973785	-0.69370488
ACH-000428	MSS	GU	M	M	-0.34344594	0.169116905	-0.633058932	2.563925031	1.166649551	0.505850282	0.662484884	-0.78355042
ACH-000430	MSS	Breast and GYN	M	M	1.427418977	0.293894837	-0.270245308	1.390908663	-1.021840349	0.469722428	-0.202739443	0.283293846
ACH-000432	MSS	Hema	M	M	-0.05732742	-1.734317233	2.01664545	-1.149567728	-2.686328331	0.117336475	0.32183801	0.228904638
ACH-000433	MSS	GU	M	M	0.248943053	-0.06379632	-0.567300102	-1.530496756	-0.680646244	0.817448746	0.440676363	-0.68796845
ACH-000435	MSS	Breast and GYN	M	M	-0.05298798	-1.001340236	0.694845784	-0.628126769	-1.215291889	1.031842186	-0.130313273	-0.63680204
ACH-000440	MSS	Hema	M	M	2.827445901	-0.168471712	0.544233815	0.262239243	-0.392669628	-2.366748598	-1.971940773	-0.62832225
ACH-000443	MSS	Breast and GYN	M	M	0.986217276	-0.945224439	-0.791931524	-0.903688144	-1.951192335	-0.069837671	-1.130455226	-0.94764768
ACH-000449	MSS	Breast and GYN	M	M	-0.64587238	1.514982491	0.985390347	0.114927853	0.135943555	1.165035449	1.847588931	-1.62827023
ACH-000459	MSS	GU	M	M	0.975478842	0.350565455	0.108279846	-0.587066435	-0.40396515	0.474939843	0.014037732	0.499940488
ACH-000460	MSS	Breast and GYN	M	W	0.437477094	0.152729642	-0.725624506	1.539441329	0.479954581	0.026751829	-0.12888492	0.236012782

ACH-000467	MSS	GI	M	M	1.031423038	0.941180024	-0.615303057	-0.03736501	-0.216927865	0.261005389	1.224704572	1.375847702
ACH-000470	MSS	GI	W	M	0.671894603	1.530398866	0.807668214	-1.869603109	-0.434583476	-0.772648821	-0.658574859	-0.27913518
ACH-000473	MSS	GU	M	M	0.837474107	0.526054605	0.883335972	-0.058982642	-1.470232158	-1.799427665	-0.020229345	-0.46125041
ACH-000486	MSS	GU	M	M	-0.31804871	-0.789340647	1.250396403	1.150403923	-2.604207164	-0.096388216	0.638514992	0.294773267
ACH-000489	MSS	GI	W	M	0.603549545	-1.361767196	-1.191781428	-0.661348413	0.073627304	0.064198463	-1.983051472	1.552255771
ACH-000500	MSS	GI	M	M	-0.59719848	-0.077808644	0.755038215	0.046548016	0.332516897	0.233561665	0.935367794	-0.76943001
ACH-000501	MSS	GI	W	M	0.821763749	0.542922109	0.398726318	-0.751687576	0.299547458	-0.355202875	1.334374721	-0.19334483
ACH-000507	MSS	GI	M	M	0.613712853	-0.76140615	-0.57112453	-1.753991609	-0.717371536	-1.788074798	-0.820989345	-0.83046437
ACH-000509	MSS	Hema	M	W	-0.02144643	0.298107243	-0.045375483	-0.456836359	0.459230014	0.55649435	-0.681740734	0.02119566
ACH-000515	MSS	other	M	M	-0.04921481	-1.576647112	-0.876873446	-2.047263824	0.135860009	1.251554314	-1.535916482	-1.69236711
ACH-000517	MSS	GI	M	M	0.308711176	-1.733013392	-2.648321128	-0.654303169	-0.220040642	-0.47012806	-1.435876059	-1.08083324
ACH-000519	MSS	Hema	M	W	-0.96818023	0.772158023	-1.211005469	-0.150832195	-0.210144991	0.977118306	-1.063416672	-0.9699318
ACH-000520	MSS	Breast and GYN	M	W	0.335183815	-0.974107552	-1.266471772	-0.629065973	-0.842955943	-0.626517923	-1.072426899	-0.43104903
ACH-000522	MSS	GU	M	M	-1.29707338	0.359097335	0.792976598	0.673273033	-0.283849596	1.083472835	-0.084604924	-0.13440576
ACH-000527	MSS	Breast and GYN	M	M	-0.19421498	-0.221210359	0.409296134	-0.637159105	-0.021311281	0.980625934	-0.454528959	-0.59203862
ACH-000532	MSS	GI	W	M	1.201663606	0.87457805	-1.185611093	0.55812918	0.294810933	-0.377003769	-0.454083687	-0.63099637
ACH-000536	MSS	Breast and GYN	M	M	0.433556701	0.020145163	-0.521239198	-0.019000525	0.48455669	0.218261997	0.68800797	-0.81748749
ACH-000542	MSS	Breast and GYN	M	M	-0.61171346	-0.221881415	-1.41850991	-0.205082823	0.017075599	0.95002043	-0.277030783	-0.39703397
ACH-000546	MSS	GI	M	M	1.301036979	-0.249448971	-0.532535847	-0.057366559	1.57125714	-0.135783421	-0.482390818	2.28666255
ACH-000552	MSS	GI	W	M	0.482539547	-0.600868259	-0.962755221	0.412297038	0.605094451	-0.048615594	-0.070996941	-0.89790691
ACH-000554	MSS	Breast and GYN	M	W	-1.42721421	-1.26036907	1.016672345	0.839243541	-2.739210323	1.363585119	0.389244125	-0.97768813
ACH-000555	MSS	GU	M	M	-0.47306978	0.384680273	-1.028672545	-0.47860623	-1.224803605	0.333145147	0.905581155	-0.46482609
ACH-000557	MSS	Hema	M	W	-0.5893109	-0.403622339	0.192317042	-0.747096757	0.353957629	-1.864345208	0.017131706	1.219501842
ACH-000560	MSS	GI	W	M	0.519372058	0.672575178	0.402933613	-0.44355591	-0.041870675	0.118944088	-0.240192977	-0.05445525
ACH-000565	MSS	GI	W	W	0.075053618	0.979675708	-1.779503111	-0.100178565	-0.934144112	-0.144819459	-0.345909777	1.080082829
ACH-000576	MSS	Hema	M	W	0.186298109	-0.173057225	-0.1553472	0.513713267	-0.031123581	1.178544845	-0.213406587	-0.43994143
ACH-000584	MSS	Breast and GYN	M	M	-1.10859845	-1.350373135	0.45721137	0.543614373	0.158524363	-0.774845041	0.667794321	-0.34526007
ACH-000598	MSS	Hema	M	M	0.025109248	-0.79298489	-1.145502914	0.165827726	0.438651179	1.336761349	-1.911256524	-2.04187849
ACH-000599	MSS	GI	M	M	-0.5154066	0.817442227	0.458665009	0.567922796	0.050389693	-0.332056764	-0.005466349	-0.69095579
ACH-000608	MSS	Breast and GYN	M	W	-0.51591325	0.791936056	-0.427278889	1.369701873	-0.999322427	0.661520134	-0.169955558	-0.3490603
ACH-000621	MSS	Breast and GYN	M	W	-0.31393725	-1.007060209	-0.351721847	0.612323103	-1.328346116	1.250316827	-2.357141961	-0.82237165
ACH-000624	MSS	Breast and GYN	M	W	0.668184182	0.939149125	0.178782711	-0.120598345	-0.381974127	-0.382085657	1.025553535	0.228280346
ACH-000643	MSS	Breast and GYN	M	W	0.238964122	0.26959317	-1.868236965	-0.380995915	0.502705304	0.016319855	0.476553872	-0.86576471
ACH-000646	MSS	Breast and GYN	M	M	-0.33353792	-0.043745726	-1.415345939	0.309207352	-2.568676344	-0.225925101	-0.215659511	0.190475608
ACH-000651	MSS	GI	W	M	0.901567175	1.166812879	1.156938483	-0.312877567	1.315483311	-0.927486883	0.356838748	-0.68818304
ACH-000657	MSS	Breast and GYN	M	M	1.362741374	0.464515791	0.521238279	0.406408984	0.285690221	-1.16641171	-1.059776952	0.772779364
ACH-000663	MSS	Breast and GYN	M	M	-0.10404966	0.38735603	-0.556265883	-0.441370001	-1.052711661	-1.135565795	-0.404977995	0.63372469
ACH-000668	MSS	Breast and GYN	M	M	-0.15903749	1.305737403	-1.244627396	-1.728300568	-0.701736488	-1.000548045	-0.180831667	0.9351126
ACH-000674	MSS	GI	M	M	1.697458466	-1.272718171	0.561138446	0.015913107	-0.672201849	-0.342468949	-0.007492855	-1.74630687
ACH-000678	MSS	GI	M	M	0.477033378	0.188943862	0.107770159	-1.694919525	-0.068022773	-0.988348553	-0.012635997	1.156637311
ACH-000679	MSS	GI	M	W	0.451961134	-0.507911645	0.63100005	-0.624297412	0.611769755	0.907251044	-0.125476675	0.058962588

ACH-000680	MSS	GI	W	W	2.142826141	1.946448173	-0.524244792	0.849786775	0.449419121	0.042205478	0.692451862	0.913990714
ACH-000683	MSS	GI	W	M	0.30195376	-0.156311357	0.434402414	-0.31431684	-0.466654193	0.66659731	-0.670941096	-0.89701052
ACH-000687	MSS	Hema	M	M	-1.32541703	-1.446275667	-0.533417816	2.369480542	-0.503254627	0.623006811	-0.116742738	-1.93303917
ACH-000693	MSS	GI	M	M	0.725110512	0.90699261	0.034098166	0.705736391	0.74879769	-0.00436012	1.067182663	-0.53336967
ACH-000696	MSS	Breast and GYN	M	W	0.912364563	-0.613651835	-0.264498348	0.214450539	-1.136156537	-0.159276372	0.20429187	-0.95835179
ACH-000701	MSS	Breast and GYN	M	M	-0.83885893	-0.225568269	0.731422892	0.92869599	-0.238842173	1.847327724	-0.152662717	0.090596208
ACH-000702	MSS	Hema	M	M	0.701904965	-1.799438204	0.470616724	0.114272988	-1.124416562	-0.252807415	-2.304566566	0.680002041
ACH-000704	MSS	Breast and GYN	M	M	-0.75386494	0.716577506	-1.465696735	-0.118746967	-0.02901404	0.018196648	0.517738491	0.290679443
ACH-000708	MSS	GI	M	M	-1.62701375	-0.142518116	-0.192214714	0.330175289	0.343561098	0.751619684	0.633610373	-0.53317591
ACH-000709	MSS	GU	M	M	-0.31419983	-0.698082759	1.890911985	-0.754222641	1.598763159	-1.429514139	-1.346073732	0.562778862
ACH-000711	MSS	Breast and GYN	M	M	0.284541595	1.796653606	0.03939709	2.317028926	1.50171945	0.383850193	1.681929626	0.496999933
ACH-000713	MSS	Breast and GYN	M	W	-0.73427363	-1.066365379	0.854740034	-0.44366305	0.46410047	-0.519084612	0.284694437	-0.66108161
ACH-000717	MSS	GI	M	M	0.582074858	0.371952878	-0.840648612	-1.211349779	2.098318822	-0.484717011	-0.440443396	-0.93580801
ACH-000719	MSS	Breast and GYN	M	M	0.266265207	-0.492828389	-0.459620118	0.532480344	-0.088672457	0.580875347	-0.336387273	-0.55806071
ACH-000720	MSS	GU	M	W	0.000575781	1.000686139	0.506279795	-2.086746804	1.042066788	0.45125771	-0.935237039	1.040231698
ACH-000721	MSS	Breast and GYN	M	W	-1.11251208	-0.828097197	0.630490334	1.022056158	2.365554826	-0.400193902	-1.722288442	1.432949649
ACH-000725	MSS	Breast and GYN	M	W	0.753718968	-0.7401697	-0.854873252	0.055956079	-0.303144159	-0.153875274	-1.408060671	-0.42450466
ACH-000735	MSS	GI	M	M	0.615908391	-0.609788465	1.216816043	0.754996981	-0.499341405	-0.734935657	-1.594826898	-1.94134237
ACH-000736	MSS	GI	M	M	0.051986348	1.304102963	-0.967740968	-0.372684634	1.088711726	0.609126685	0.59902636	0.403729412
ACH-000740	MSS	GI	M	W	-0.59180613	0.573442508	0.697805771	-1.567508047	0.701274788	-1.27302199	-0.184373131	0.174683064
ACH-000746	MSS	GI	W	M	-1.92560691	-1.364560454	0.431447649	-0.14004	-0.932008383	0.852265622	0.648487972	-0.71221724
ACH-000747	MSS	other	M	W	0.754111171	-0.271764087	2.136662164	-0.799056981	-0.36523505	-0.011776529	0.783119554	-2.12594344
ACH-000751	MSS	Hema	M	W	-2.06083818	0.475286068	-0.504498253	-0.035661365	-0.080843646	1.180260435	0.581093931	0.306683244
ACH-000758	MSS	GI	W	M	0.864762236	-0.298250568	-0.431197942	-0.959434256	-0.601706135	-2.253064805	0.505969061	-0.18362447
ACH-000759	MSS	Breast and GYN	M	M	1.674545297	-0.28167182	-0.521622705	0.052424357	0.611448659	-0.486449501	-1.985259592	-1.27826169
ACH-000762	MSS	GI	W	W	-0.43801862	-0.127936534	-1.335768871	0.058910766	-1.125511784	-0.267014379	0.589156673	-0.01106385
ACH-000765	MSS	other	M	M	1.086004719	0.841806851	-1.321409539	-1.881765171	-0.212651746	-2.806311555	1.090468094	0.819565266
ACH-000768	MSS	Breast and GYN	M	M	1.147819508	-0.04199542	0.652126327	-0.358257158	-0.501997189	0.40603287	-0.554045369	-2.067024
ACH-000769	MSS	other	W	M	1.129024311	-0.578044942	-0.173446322	0.378238632	-0.325866365	-1.425754476	0.115681103	-0.86047439
ACH-000770	MSI	Hema	M	W	-2.56956011	0.312691843	-1.190870612	0.14085214	-0.811920944	0.106195438	0.946868151	-0.41401622
ACH-000773	MSS	Hema	M	M	0.602915934	-0.886759645	-1.275509522	-1.418463598	-0.204891244	-0.814847204	-1.808351623	-0.61736616
ACH-000782	MSI	Hema	M	W	1.314476224	0.121411871	-2.077174306	-0.860038183	0.328801927	0.545449642	-0.727465334	1.541286475
ACH-000783	MSS	Breast and GYN	M	M	0.748279916	-0.235967243	-0.171364554	1.066136294	-0.950308157	0.169912865	-0.058405276	1.387046789
ACH-000792	MSS	GU	M	W	-0.57735291	1.22362933	-0.806027346	-0.923063819	-0.308958703	-0.776913108	0.409429402	0.619019669
ACH-000798	MSS	GI	W	M	1.101654884	-1.788706306	-0.558613579	-0.893731598	-0.964245644	-0.894610858	-1.512059311	-0.56275562
ACH-000808	MSS	GI	M	M	0.823452007	-0.468675299	0.348605073	-0.09924021	0.268583484	-0.180577468	-0.220957413	-1.28281323
ACH-000810	MSS	other	W	W	0.17471282	-0.645106144	-0.375801603	-0.163686971	0.5908217	-0.882450535	-0.647931341	-0.76415983
ACH-000811	MSI	Breast and GYN	M	W	0.502553668	1.423732808	-0.378264908	0.912934792	0.321814851	0.698718106	0.929490128	0.745274448
ACH-000820	MSS	GI	W	W	1.282186691	0.798336179	0.038699434	-0.258065604	-0.815192817	0.67066213	0.802054824	-0.43139315
ACH-000824	MSS	GI	M	W	-0.12094134	0.613619932	-1.193819762	-0.786468594	-0.73987764	0.533421402	0.76869129	1.007853165
ACH-000828	MSS	Breast and GYN	M	M	0.494072042	-0.456755943	-0.19380378	0.21211745	-1.49476647	-0.136596905	-0.779301158	-2.85533684

ACH-000831	MSS	Breast and GYN	M	M	0.831306977	0.401247848	0.674128289	0.378637911	-1.021266915	-0.42535295	-0.622785669	0.296279797
ACH-000832	MSS	GI	M	M	0.171323003	-1.058906562	-1.048788779	1.277087389	-0.920626526	-0.136624313	0.464180729	-0.90848266
ACH-000842	MSS	GI	W	M	0.128708131	1.457177304	-1.240621887	1.847466673	0.684776084	-0.287485924	1.115565429	0.988825885
ACH-000847	MSS	GI	W	W	0.109380212	0.292604428	0.795509025	0.275457914	-0.690404889	-3.009763793	0.91352179	1.648843646
ACH-000849	MSS	Breast and GYN	M	M	-0.8899338	0.067827886	-0.921166282	0.408412408	0.020994911	1.617768957	-0.264836922	-0.86917971
ACH-000856	MSI	Breast and GYN	M	M	0.691022578	0.330773796	1.71123609	0.057818014	0.118844096	-0.348758342	1.035948787	-0.33041954
ACH-000859	MSS	Breast and GYN	M	M	-0.88873196	0.316722446	-0.7467323	0.84717593	-0.016959148	1.788348078	0.833277379	-0.36461372
ACH-000862	MSS	GU	M	M	-1.26903294	-1.093597046	0.145666374	-0.382438656	-1.784472993	-0.152346612	-0.581785253	-0.30594726
ACH-000864	MSI	Breast and GYN	M	W	-1.20294049	0.445876973	1.26358281	1.553633215	-0.00310233	2.257799394	2.062368623	1.441310602
ACH-000866	MSI	other	M	W	-0.34707798	0.621267947	2.329753686	-0.686101538	-0.649062706	0.256027264	0.873230626	-0.06148282
ACH-000874	MSI	Hema	M	M	-1.20581762	0.632143762	1.519134582	-1.206374804	2.516063683	0.761088228	0.263510726	1.429695432
ACH-000879	MSI	Breast and GYN	W	W	-1.32477585	1.508518939	0.593260176	0.658731687	0.106002572	0.932543445	0.942490871	0.683258164
ACH-000885	MSI	Breast and GYN	M	M	-1.05436019	-1.243479196	-0.782717999	1.376014122	0.958344025	0.382457515	-1.036543175	1.49001696
ACH-000895	MSI	GI	W	W	0.732159783	0.907217755	1.397914141	0.940392179	0.310826494	0.134952687	0.339074445	1.911172468
ACH-000897	MSI	other	M	M	-0.64508148	-0.504957836	-0.247451738	0.202706913	0.34898752	0.490553903	-0.209342835	-1.06721483
ACH-000898	MSS	GI	M	M	0.384187273	0.981674766	-0.514234962	-0.246424175	0.786670412	-2.80711476	0.302029671	0.318365978
ACH-000899	MSS	other	M	M	0.850095301	1.350062528	-0.670482463	1.088223474	1.174530724	0.749767978	0.502283014	0.55051895
ACH-000906	MSS	Breast and GYN	M	M	-0.3473728	1.652511163	-1.044596817	-2.272880466	0.721717661	-0.000889018	0.907383404	-1.09951638
ACH-000907	MSI	GU	M	M	-0.47577701	1.134918678	0.956529286	-0.875368428	0.760225627	-0.372101698	-0.501767705	-0.82032866
ACH-000908	MSI	GI	M	M	-1.18203933	-1.499405284	2.037296032	0.15498587	0.677838587	1.880164239	-0.350550638	-0.4529821
ACH-000909	MSI	Breast and GYN	M	M	-1.5779038	-0.485594531	-0.091063683	-0.475745103	1.182207248	0.528960505	-0.182020244	0.535257798
ACH-000910	MSS	Breast and GYN	M	M	0.761572671	0.252164205	-0.648121551	-0.996192699	0.892810954	0.832033878	0.463431032	2.080277637
ACH-000911	MSI	GI	M	M	-0.90813781	0.069691693	2.337442223	0.663291193	0.75038832	1.300928104	-0.059929506	1.449140225
ACH-000913	MSS	Breast and GYN	M	W	0.804686074	0.497679119	0.042769223	-0.022791112	-1.098921121	0.197698255	-0.32590896	-0.23542064
ACH-000914	MSI	Hema	M	M	-0.61156274	0.057089229	0.386034279	-0.059650799	0.65554195	-1.808143937	0.880072196	1.47307283
ACH-000919	MSI	GI	M	M	0.648685225	-0.949005113	0.170306845	0.233931131	0.180429764	1.388017076	0.354910706	0.319916248
ACH-000920	MSI	Hema	M	M	0.288353622	-0.163399332	-1.127914396	1.594443418	0.563320156	1.234332638	0.551834604	1.636421083
ACH-000921	MSS	other	M	W	0.47380825	-0.182667026	-0.625527608	-1.492604921	-1.02186179	-1.035692808	-0.407330549	-1.15413019
ACH-000925	MSI	other	W	M	-0.51464027	-0.286132055	0.196466043	0.375450786	0.67264583	1.701262212	1.28443098	-0.39706325
ACH-000926	MSS	GI	W	M	-0.03952792	0.772784336	-0.403879077	-0.276227014	0.047376457	-0.122072279	-0.43396156	-0.35807272
ACH-000927	MSS	Breast and GYN	M	M	0.165930184	-1.312798204	-0.173100754	-0.047898379	-1.018001208	-0.725366697	-0.805395768	-1.34559618
ACH-000928	MSI	Breast and GYN	M	M	0.91566821	0.082466474	0.9432282	-0.206334479	1.005692048	0.781015411	0.259026768	0.036485214
ACH-000930	MSI	Breast and GYN	M	W	-1.02644059	1.864878922	-1.242478817	1.082015875	-1.68974157	0.894770553	-0.385235395	1.800083503
ACH-000932	MSI	GI	M	M	-1.31829241	0.556828757	-1.916210779	0.513706907	0.87769646	-0.466342217	1.070046579	0.764656034
ACH-000933	MSI	GI	M	M	-1.11030763	0.140909169	2.519550421	0.756036254	2.594357347	0.133765995	1.257457383	-0.26802321
ACH-000934	MSS	Breast and GYN	M	W	0.790005688	-2.717632275	0.091458687	0.425162971	0.277596609	-0.666510745	-1.734268404	-1.73770039
ACH-000935	MSS	GI	W	M	0.031612926	0.609813344	-0.926548681	1.301640128	0.88110968	-0.933268108	-0.093422454	-0.09415489
ACH-000936	MSI	Breast and GYN	M	W	2.019216734	-2.067719613	0.766738203	-0.416510032	-0.501746935	-0.876911454	-1.055496707	0.81426952
ACH-000937	MSS	Hema	W	M	-0.15459723	0.650952658	0.663384233	-1.278468146	-0.80640526	-1.055422366	0.129313265	0.127609553
ACH-000938	MSI	Hema	M	M	-1.49821235	0.627993986	0.797240746	2.048271203	0.746768566	0.073573266	0.74392675	1.320848002
ACH-000939	MSI	other	W	M	-0.62035853	-0.430729831	0.984256044	0.986191114	0.323847016	0.574436936	-0.185226916	0.844666277

ACH-000940	MSI	Breast and GYN	M	W	-1.074595	0.507739919	0.495812453	-0.647040567	-0.654462823	0.843212241	0.08432846	0.007424022
ACH-000941	MSI	Breast and GYN	M	M	-0.91002057	1.759379718	1.096571705	1.177943239	0.311241762	-0.303027025	2.660333063	1.091220353
ACH-000942	MSI	Hema	M	W	-1.64064888	1.128791445	2.36184856	0.212973908	1.516465154	1.057035093	0.786736401	0.548536085
ACH-000943	MSI	GI	M	M	-0.17479537	0.464723366	1.783166688	-0.495663669	-1.016695674	0.685574332	1.210481467	0.381312597
ACH-000944	MSS	Hema	M	M	1.19455883	-0.479325956	0.587761122	-0.160780873	-0.90440276	1.167650478	-0.337778021	0.120809071
ACH-000946	MSI	Breast and GYN	M	M	-0.37075525	-0.67262415	0.730499188	1.86971494	-1.076204743	1.235460168	0.574611161	0.867985216
ACH-000947	MSI	Breast and GYN	M	W	1.373115516	-0.224816594	0.165239292	-0.616008972	0.213882274	0.251235584	-1.979524955	0.622079013
ACH-000948	MSI	GI	M	M	-0.00791189	1.893575835	1.640037795	-0.360102678	0.973117637	-0.707575947	0.539516609	2.214681427
ACH-000949	MSI	GI	W	W	-1.8993709	-0.769369619	1.304949608	1.232433605	0.435745239	-0.592592519	0.518002468	1.416177411
ACH-000950	MSI	GI	W	M	0.291796674	-0.042500982	0.801281387	2.001776719	-0.311130159	0.2345549	0.032104399	0.130600315
ACH-000952	MSI	GU	M	M	-1.4868661	-0.251120967	-1.137152352	-0.49906698	-0.191701281	2.918107158	0.856500044	-0.03670101
ACH-000953	MSI	Hema	M	M	-0.38105256	1.013298597	0.62446013	-0.4550832	0.610691847	1.085193735	-0.11676357	0.335932636
ACH-000954	MSI	Breast and GYN	M	M	0.438382399	1.003440073	-1.550506658	-0.549076448	1.027481986	-0.362523654	0.96122215	1.443852649
ACH-000955	MSI	GI	M	W	0.433898595	2.324221955	-0.751596897	-0.172198107	1.666547163	-0.680083607	0.843321187	1.337006667
ACH-000956	MSI	GU	M	W	-0.2961314	-0.069283215	0.451467775	0.532029238	-0.760657648	1.640348909	0.933997912	0.980575924
ACH-000957	MSI	GI	M	M	-3.81882783	2.317785357	-1.950403419	3.105143602	-1.024802017	0.486871678	1.093050411	-0.18532309
ACH-000958	MSI	GI	M	M	0.561090408	0.276866338	1.187258917	-0.656405024	1.438413876	-0.276764876	-1.268089182	0.977965024
ACH-000959	MSI	GI	W	M	-0.20863412	0.356216949	0.410093925	2.285889268	0.216469599	0.414977468	0.054226681	0.827247283
ACH-000960	MSI	Hema	M	M	-1.24485492	-1.035075117	-0.27333996	2.167769115	0.175502002	0.517881539	1.057274105	-1.38089309
ACH-000961	MSI	Breast and GYN	M	M	0.438039424	0.345754027	1.673118602	0.638793781	1.53202722	-1.166126681	0.045628873	1.571399542
ACH-000962	MSI	Breast and GYN	M	M	-0.49287115	1.070411123	0.239396103	-0.615541466	1.618086356	-0.488775197	-0.037382158	0.834972207
ACH-000963	MSI	GI	M	M	1.588086912	-1.953922504	-0.856290481	-1.920743332	0.010037646	0.132396402	-1.646916982	-1.94580012
ACH-000964	MSI	Hema	M	M	-2.23359079	-0.318790101	0.084372194	0.63172004	1.226214803	-0.876372123	-0.010321849	0.300846883
ACH-000965	MSI	Breast and GYN	M	M	-2.03092291	-0.431216747	1.45753183	-0.363565511	0.053460667	1.056201313	0.838212106	-0.57909534
ACH-000966	MSI	Breast and GYN	M	W	-0.46727208	1.53434423	0.877261718	-0.40890699	0.339978078	0.278580552	-0.196688509	0.579723875
ACH-000967	MSI	GI	W	M	-0.03997399	-0.260130606	-0.586635241	0.696060547	-0.12473119	-0.010255592	1.704099357	1.006893152
ACH-000969	MSI	GI	W	W	0.88809129	1.450225371	-0.72346151	0.100039558	0.672782901	0.322522291	0.381343801	0.716155843
ACH-000970	MSI	GI	W	M	0.394407443	1.025621614	-0.238625212	0.756230714	2.743742022	0.434721865	0.357689959	0.37504716
ACH-000971	MSI	GI	M	M	-0.717164	-0.116730169	2.750892055	0.852108434	-1.388251659	0.272482001	0.768658103	1.305887952
ACH-000972	MSI	Breast and GYN	M	M	-0.15496729	0.820483698	1.324565655	1.373792168	-0.313751716	0.707437982	1.375644669	1.481734095
ACH-000973	MSI	GU	M	M	-0.65622362	0.240897372	0.342700183	0.377690364	0.709893157	-1.019908852	0.846526461	1.990035031
ACH-000974	MSI	Breast and GYN	M	M	0.675613929	1.022777475	0.662718285	-0.803588494	0.32417565	-0.185587304	-0.981659276	0.985885404
ACH-000977	MSI	GU	M	M	-1.45483408	1.588622999	0.10308203	-0.086243163	1.105818839	1.444011823	0.785681845	-0.04539187
ACH-000978	MSI	Breast and GYN	M	W	-0.77500593	1.891563437	0.564715003	0.735120273	0.206392007	0.69045305	1.44102358	-0.26673964
ACH-000980	MSI	other	W	M	0.088357364	2.859143322	0.704915556	0.736955374	2.294501537	0.976360671	2.558125758	-0.46091347
ACH-000981	MSI	Hema	M	W	0.152397089	0.056599416	0.745750258	0.413009345	0.915118729	1.155570671	0.77604093	0.512809839
ACH-000982	MSI	GI	W	M	0.308534762	1.673574897	-1.55814222	0.483302876	1.032750214	1.167798675	1.659964061	0.911522663
ACH-000983	MSI	Hema	M	W	0.39278586	0.719316561	0.964052831	0.485415161	-0.429028806	0.242699286	-0.007694953	-0.65608277
ACH-000984	MSI	Breast and GYN	M	M	-1.07500416	1.710726077	-0.082924564	-0.713403673	0.537629576	1.758816043	1.36273859	0.398501257
ACH-000985	MSI	GI	W	W	-0.51529717	1.475919235	1.442052065	0.928528196	1.217027897	-0.341776697	1.64069536	1.682963484
ACH-000986	MSS	GI	W	W	1.286004586	-1.005212524	-0.244932713	-0.103893895	1.765030707	0.152320117	-0.838973456	0.146579344

ACH-000988	MSI	Breast and GYN	W	M	0.108371174	1.43459637	-0.577819263	1.547301698	1.360212633	0.49931841	0.384263673	1.449335495
ACH-000989	MSI	GI	W	M	-1.61076296	-0.598934878	-0.207630766	2.154673086	-0.532982821	-0.014447419	2.45240755	1.717492832
ACH-000990	MSI	Breast and GYN	M	M	0.51797065	-0.279025599	0.752079115	0.193214526	0.18275966	0.106515769	0.395281236	0.967129925
ACH-000991	MSS	GI	W	W	-0.15639387	1.419295293	0.553002322	0.555255776	0.24592182	0.084901359	0.836136038	-1.48827238
ACH-000993	MSS	Breast and GYN	W	M	-0.06426476	-0.360068333	0.944520803	-0.33272866	-0.495708428	-0.886250988	0.188613181	0.396592381
ACH-000994	MSI	Breast and GYN	M	M	-0.8926087	-0.063778031	-0.243812432	0.305496562	-0.102564157	0.182474709	0.321288677	1.771691421
ACH-000995	MSI	Hema	W	W	-0.89482001	-1.484359319	-0.38324731	2.244499647	1.669239833	0.428419766	0.552823658	1.299922385
ACH-000996	MSS	Breast and GYN	W	W	-1.22422201	-0.633119386	2.290604227	-0.236992845	0.091852953	0.818691244	-0.411607334	-0.82983204
ACH-000997	MSI	GI	W	W	-0.72369682	0.764713572	-0.076702268	0.80300918	-0.522244477	0.361647654	-1.178234156	2.067311709
ACH-000998	MSI	GI	W	M	-1.45419044	0.01543148	0.82339944	1.603523503	2.445297856	1.607967852	0.09369274	-0.20732365
ACH-000999	MSI	GI	W	W	0.124480247	-0.242800099	-1.12926534	-0.858453208	-0.021402467	0.479193013	-0.847598156	0.100228598
ACH-001145	MSI	Breast and GYN	M	M	-1.104028	0.93251584	1.106872032	-0.289586848	0.471745462	0.059627857	0.95688466	2.404757505
ACH-001151	MSS	Breast and GYN	M	M	0.696363139	-1.972520933	-0.779057148	-0.011447681	-1.046295727	1.645205111	-0.709751764	-2.64361609
ACH-001190	MSI	other	M	M	-2.26191676	0.438912948	1.368024463	-0.425986455	-0.681572337	1.76939768	1.1294047	-0.77112287
ACH-001321	MSS	other	W	M	1.536959991	-0.878892405	0.194092202	-0.522576644	0.488224782	0.104979116	-1.654328925	0.126654573
