# Supplementary material for: Controlling the confounding effect of metabolic gene expression to identify actual metabolite targets in microsatellite instability cancers
Source: Hum Genomics. 2023 Mar 6;17:18. doi: 10.1186/s40246-023-00465-9 (PMC9990231; doi:10.1186/s40246-023-00465-9)
Supplement: Supplementary file 8 — Additional file 8: Table S5. Relationship between adjusted metabolite features and metabolic genes. [file 40246_2023_465_MOESM8_ESM.pdf]

Supplementary Table S5. The relationship between adjusted metabolite features and metabolic genes

Metabolites	Hippurate	3-phosphoglycerate	C14:0 CE	C18:0 LPE	6-phosphogluconate	C36:1 PC	Glutathione reduced	Sarcosine	Pathway
GALC	-0.031257976	0.026122683	-0.013779505	-0.008801181	0.003335772	0.021840915	0.010971394	-0.011957732	Metabolism of lipids
CERS4	0.081984304	0.025299883	-0.059858735	0.02771255	0.039015138	-0.097415725	0.060959324	0.055732371	Metabolism of lipids
PTGS1	-0.001161727	0.012317411	0.032211872	0.093379961	-0.01215517	0.075355428	-0.029246192	0.088900957	Metabolism of lipids
DHCR24	0.064051644	0.014990855	0.032299996	-0.074607806	0.001113694	-0.029574241	-0.03377386	0.082719616	Metabolism of lipids
CBR1	-0.039419742	-0.077715823	-0.016170277	0.01662755	-0.049076498	0.048428417	-0.027517447	-0.070197058	Metabolism of lipids
GPX2	0.008342249	0.079688762	-0.017900171	0.011310542	0.049382955	-0.007302349	0.053928529	0.075245537	Metabolism of lipids
CYP51A1	-0.090699586	-0.005797981	0.040356965	-0.037206098	-0.052249701	0.160786049	0.080861057	0.019176765	Metabolism of lipids
AGPS	-0.042865316	0.02477576	0.194995827	0.030936993	-0.102456446	0.006530936	0.057070849	0.115376298	Metabolism of lipids
PLD1	0.072855856	0.048049377	-0.064165638	-0.040050787	-0.01163694	-0.015330063	0.052773783	0.123921022	Metabolism of lipids
PLA2G3	0.046107901	0.096953208	0.011943759	-0.150712505	-0.207245588	-0.208855873	0.010086824	0.190713472	Metabolism of lipids
CERK	-0.0131892	0.051970206	-0.052123036	0.009640986	-0.023732034	0.025084141	-0.007948994	0.15119483	Metabolism of lipids
SQLE	-0.073001246	0.000755312	-0.15309546	0.005258171	0.092730571	-0.072018136	0.035890508	-0.133413736	Metabolism of lipids
SC5D	-0.018235269	-0.162512769	-0.072161681	0.08942545	-0.126592017	-0.052453133	0.069263458	-0.06937909	Metabolism of lipids
CHPT1	-0.113727237	0.070588138	-0.030703036	0.089893096	-0.02384358	-0.029101357	0.092468438	-0.014071101	Metabolism of lipids
EPHX2	-0.078799714	-0.041144188	-0.105760702	0.038859406	-0.078950851	0.041199805	-0.02817371	-0.03191746	Metabolism of lipids
SGPP1	0.211416084	0.062265237	0.093519468	-0.040855788	0.009442461	-0.033650506	-0.036953813	0.110221589	Metabolism of lipids
CERS6	0.047867406	-0.066101319	0.066708785	0.011898537	-0.047319537	0.129220875	0.07424577	-0.022378988	Metabolism of lipids
AKR1B1	0.021789383	-0.046299709	-0.043081021	-0.013031506	0.019045759	-0.005903088	-0.039275691	-0.022456399	Metabolism of carbohydrates
XYLT1	-0.03676037	-0.062675405	-0.006169851	-0.058534974	0.039606002	0.026667097	-0.043729145	-0.003911137	Metabolism of carbohydrates
ALDOC	-0.051261958	-0.028993613	0.015994104	-0.026128706	-0.007574341	0.051497366	0.008069239	-0.075876941	Metabolism of carbohydrates
ENO2	0.020046629	-0.002167273	-0.08931803	0.017449613	0.048414179	0.006791521	0.067714017	0.053221199	Metabolism of carbohydrates
B4GALT6	-0.057437185	-0.061730645	0.088614275	-0.016606012	0.033713309	0.038268793	-0.065947633	-0.089748978	Metabolism of carbohydrates
CHPF	0.087748759	0.031310515	0.018916497	-0.079408224	0.024557297	-0.052127373	-0.003912459	0.040740154	Metabolism of carbohydrates
HS3ST3B1	0.011812624	-0.032551886	0.025286319	0.070446886	0.007966925	0.049547777	0.019444351	-0.061676942	Metabolism of carbohydrates
HS6ST1	-0.019095611	-0.053064259	-0.012848681	-0.036053567	0.041912844	-0.046287927	0.00320961	-0.085434871	Metabolism of carbohydrates
PC	-0.098473083	0.083690427	-0.008128326	0.002307719	0.07528978	0.067057882	0.079798172	-0.007681608	Metabolism of carbohydrates
B3GNT3	0.034840639	0.049609819	-0.038620697	-0.002842434	-0.008073236	0.014462949	0.059551861	0.040520333	Metabolism of carbohydrates
SGSH	0.004423389	0.060813765	0.067196833	0.066244258	-0.036595602	-0.012731929	0.041298308	0.008449146	Metabolism of carbohydrates
HS3ST1	-0.027250833	0.094847456	0.056436848	0.123439112	0.067601774	0.100524402	0.062741354	-0.121857079	Metabolism of carbohydrates
SLC25A13	0.070537431	0.056876831	-0.169180045	-0.024448602	0.115603456	-0.115491746	0.029224247	0.005015458	Metabolism of carbohydrates
HEXB	0.157150331	0.001817028	-0.066725831	0.077548263	-0.073357144	-0.090525952	0.013818082	-0.1641628	Metabolism of carbohydrates
PFKP	-0.023519319	0.133069504	-0.05501531	0.084199803	-0.030485341	0.01044241	0.095767388	0.080985086	Metabolism of carbohydrates
PGM1	-0.03833377	-0.074403097	0.132955807	-0.061965453	-0.035158165	-0.040654265	-0.050374503	-0.109305565	Metabolism of carbohydrates
XYLB	0.109036324	0.088501521	-0.020086349	0.037892458	-0.105158876	-0.037762003	0.033557119	0.003986888	Metabolism of carbohydrates
GALK1	-0.114904015	-0.007242718	-0.028927481	-0.128705892	-0.046919321	-0.007685731	-0.136896899	0.102828676	Metabolism of carbohydrates
GNPDA1	-0.023721372	0.092592243	0.095445494	-0.101827556	0.039931637	-0.031970325	-0.081066622	0.014463491	Metabolism of carbohydrates
B4GALT2	0.148435204	0.011319127	0.166737012	0.110151852	0.242206792	0.194740222	-0.019068764	-0.083137064	Metabolism of carbohydrates
PPP1R3C	0.073898525	0.087565266	0.03676417	-0.082032475	0.127964778	-0.018515904	-0.046936472	0.114669977	Metabolism of carbohydrates
CHST3	-0.042803821	0.023359322	0.013332455	-0.125937842	-0.059937515	-0.047719087	-0.013290015	0.081131256	Metabolism of carbohydrates
PFKFB2	0.016952467	0.081069633	0.10325951	-0.085935517	0.183890828	-0.054451131	-0.013021692	-0.068694717	Metabolism of carbohydrates

**IDUA**	0.006740069	-0.068065197	-0.007505347	-0.058923129	-0.127928035	-0.094959292	-0.03164328	0.035507874	Metabolism of carbohydrates
**HK1**	0.01824152	0.030381617	-0.119889836	0.057842413	-0.048021601	0.02117897	0.006391797	-0.066990945	Metabolism of carbohydrates
**ST3GAL2**	-0.082569414	0.154965811	0.057857678	0.005119402	0.009350825	0.019729823	0.123933902	-0.068428298	Metabolism of carbohydrates
**HK2**	0.054698929	-0.052828357	-0.032477958	-0.050155721	0.040933919	-0.120135818	-0.242543348	-0.01187488	Metabolism of carbohydrates
**GYG1**	-0.001439777	0.023609323	0.148210083	-0.016242677	-0.020511125	-0.095435594	0.00744545	0.233719848	Metabolism of carbohydrates
**CRYL1**	0.030280039	0.039969653	-0.029039946	-0.132770544	0.032087056	0.010648035	0.007939086	0.106196942	Metabolism of carbohydrates
**HPSE**	0.005687375	-0.12085739	0.016020328	-0.058749011	-0.10127531	-0.044423497	-0.008557206	-0.123742658	Metabolism of carbohydrates
**CHST2**	0.024914049	-0.073060871	-0.034597371	-0.04160593	0.075205666	0.11082052	0.015034245	-0.094508372	Metabolism of carbohydrates
**SMOX**	-0.036699907	0.003887154	0.014727049	0.031879673	0.014208627	-0.005221475	0.043382129	0.023301042	Metabolism of amino acids and derivatives
**PHGDH**	0.048425481	0.029087765	0.048118442	-0.028856617	-0.045150021	-0.003220068	0.018034481	0.040294121	Metabolism of amino acids and derivatives
**GAMT**	0.035560936	0.070307696	0.047109487	-0.010081436	0.047699514	0.021009178	0.069681821	0.09109068	Metabolism of amino acids and derivatives
**ASS1**	0.004494302	0.025182485	-0.041719622	0.022568892	0.022503193	-0.003981951	0.019027626	0.0332435	Metabolism of amino acids and derivatives
**BHMT2**	0.02835514	0.003949917	-0.07073044	-0.084885933	-0.024862516	-0.016046757	-0.006058023	-0.039471947	Metabolism of amino acids and derivatives
**PSAT1**	-0.001004059	-0.005894836	-0.02484797	-0.057460254	-0.015935969	-0.083022429	-0.015185854	-0.011279313	Metabolism of amino acids and derivatives
**GLUL**	0.027700973	-0.089841424	0.000572494	-0.017527613	-0.08825415	0.007182731	-0.040760461	-0.024788323	Metabolism of amino acids and derivatives
**CBS**	-0.014036053	0.03266795	0.030391784	0.055466631	-0.00380411	0.00711558	0.048618048	0.00832901	Metabolism of amino acids and derivatives
**ALDH7A1**	0.08956106	0.031376573	0.063257806	0.011001725	0.028230045	-0.000278718	-0.002332202	0.022631617	Metabolism of amino acids and derivatives
**CKB**	-0.009545219	-0.078862063	0.029816164	-0.043499331	-0.028697299	-0.015395059	-0.034377188	-0.046470572	Metabolism of amino acids and derivatives
**NQO1**	0.007749108	-0.016236355	0.076423295	-0.005856276	0.01657383	-0.040623791	0.022775554	-0.012599192	Metabolism of amino acids and derivatives
**AASS**	0.0331837	0.03816744	-0.024996693	0.131865641	-0.076540534	0.056509186	-0.000860536	0.068340056	Metabolism of amino acids and derivatives
**CHDH**	-0.033437438	-0.157080435	0.067720638	0.129851539	-0.001003653	0.086864603	-0.265429339	-0.07231709	Metabolism of amino acids and derivatives
**MRI1**	-0.059238565	-0.048392847	-0.041134676	0.043608471	-0.116781475	0.048788728	0.098703564	0.073313481	Metabolism of amino acids and derivatives
**OAT**	-0.093357314	0.046411975	0.105593136	0.006488679	0.094537296	0.01705346	-0.090715602	-0.007993245	Metabolism of amino acids and derivatives
**ASNS**	0.072704888	-0.158390067	-0.009120797	0.037087483	-0.054226553	0.030872401	-0.072763219	-0.045209796	Metabolism of amino acids and derivatives
**SLC7A5**	-0.103480818	-0.039268081	-0.033539247	0.043944696	0.00282271	0.082691146	0.05725159	-0.055843089	Metabolism of amino acids and derivatives
**FAH**	0.124429354	-0.04467962	-0.11499097	0.046193973	0.150918761	0.120841876	0.097272136	-0.068028914	Metabolism of amino acids and derivatives
**AADAT**	-0.00658204	0.001259609	-0.060971873	-0.007036275	0.056697826	0.019147029	-0.029365715	-0.127752185	Metabolism of amino acids and derivatives
**HGD**	-0.009128769	-0.035388149	-0.021693054	0.009609416	-0.145171438	-0.036672639	0.032070267	-0.007905668	Metabolism of amino acids and derivatives
**AGMAT**	0.058266486	0.011175268	-0.069472976	0.024757965	-0.015715677	0.056988683	0.02950462	0.116842759	Metabolism of amino acids and derivatives
**ASL**	0.052375224	-0.155558889	-0.19444739	-0.053691748	0.085535985	0.148467947	-0.055149899	-0.172924211	Metabolism of amino acids and derivatives
**MPST**	-0.209305667	0.018300281	0.029853536	0.107550174	-0.023400359	0.026312354	0.046039597	-0.114958135	Metabolism of amino acids and derivatives
**IVD**	0.003289256	-0.012145851	0.044180868	0.050848001	0.110500164	-0.020641699	-0.039076523	-0.022584853	Metabolism of amino acids and derivatives
**CDO1**	0.051613866	0.000105295	0.077696269	-0.11162991	0.017441449	-0.109486715	-0.131688106	-0.09925957	Metabolism of amino acids and derivatives
**PAOX**	0.100549034	-0.132785742	-0.083455741	-0.243653275	-0.026477875	-0.033142696	-0.36791417	-0.038465418	Metabolism of amino acids and derivatives
**QDPR**	0.182818278	-0.203349659	-0.026526164	0.003462352	-0.183313176	-0.010780411	-0.070968193	0.013666798	Metabolism of amino acids and derivatives
**ALDH4A1**	0.048450039	0.099969624	-0.052095813	-0.022288701	-0.027349381	-0.106953524	0.047521487	0.181647552	Metabolism of amino acids and derivatives
**GPT2**	0.041048306	0.074779814	-0.07275508	-0.059409977	-0.025556363	0.047660417	0.011266771	0.136008271	Metabolism of amino acids and derivatives
**SLC36A4**	-0.084664565	0.088780533	0.039664341	0.076992275	0.115433665	0.045389731	0.064098766	0.065556462	Metabolism of amino acids and derivatives
**PYCR1**	0.023670839	0.022744767	-0.021863131	0.014001594	0.107281263	-0.044864918	0.066259259	0.036841298	Metabolism of amino acids and derivatives
**NAT8L**	0.009712124	0.024525234	-0.011501037	-0.053457845	-0.137011837	0.015849259	-0.041293326	-0.08324503	Metabolism of amino acids and derivatives
**NME3**	-0.046957904	-0.063804854	-0.051022539	0.061134194	0.017385299	0.085176081	0.021924229	-0.020241588	Metabolism of nucleotides
**NME4**	-0.064203254	-0.041564004	-0.004708403	0.033558694	-0.029552079	0.047398271	-0.022933646	-0.081516243	Metabolism of nucleotides

**GDA**	0.066522375	0.092099815	-0.035066575	-0.036921843	0.017863462	-0.017907428	0.015448382	0.060293388	Metabolism of nucleotides
**NT5C**	0.045192598	-0.001710373	0.083190299	0.014930729	-0.005217159	-0.072611346	0.067824114	0.073834888	Metabolism of nucleotides
**DCTD**	0.041651852	0.006629208	-0.172339551	0.090475869	-0.091820661	0.029506881	0.110830154	0.087305334	Metabolism of nucleotides
**TK2**	0.063425957	-0.166072805	-0.103621323	-0.13238494	-0.003616862	-0.00443602	-0.085820298	-0.288080568	Metabolism of nucleotides
**ENTPD3**	-0.152404995	0.09597816	0.156270598	-0.058033553	0.00464772	0.017155085	0.100030716	0.083652285	Metabolism of nucleotides

CE: cholesterol ester
LPE: lysophosphatidylethanolamine
PC: phosphatidylcholine
