# Supplementary material for: Controlling the confounding effect of metabolic gene expression to identify actual metabolite targets in microsatellite instability cancers
Source: Hum Genomics. 2023 Mar 6;17:18. doi: 10.1186/s40246-023-00465-9 (PMC9990231; doi:10.1186/s40246-023-00465-9)
Supplement: Supplementary file 9 — Additional file 9: Table S6. Comparison of fold change in sarcosine between microsatellite instability cancer patients and healthy controls. [file 40246_2023_465_MOESM9_ESM.pdf]

Supplementary Table S6. Comparison of fold changes in sarcosine between MSI cancer patients and healthy controls

Patients	Diagnosis	Post-operative	Follow-up	Cancer recurrence
1. CRC	NA	1.9	3.5	Yes
2. CRC/EC	NA	3.4	NA	Yes
3. CRC	2.4	NA	NA	No
4. EC	2.9	0.64	1.1	No

Abbreviations: CRC, colorectal cancer; EC, endometrial cancer; NA, not available.

Supplementary Table S6. In MSI patients with recurrence, the sarcosine level exhibited 1.9- to -3.5-fold increases during the postoperative and follow-up periods (Supplementary Table S6 and Supplementary Fig. S3). In MSI patients without recurrence, the sarcosine level exhibited 2.4- to 2.5-fold increases at the time of diagnosis and no fold change during the postoperative and follow-up periods.
